# Lifestyle, WCRF/AICR Recommendations, and Esophageal Adenocarcinoma Risk: A Systematic Review of the Literature

**DOI:** 10.3390/nu13103525

**Published:** 2021-10-08

**Authors:** Daniele Nucci, Alessio Marino, Stefano Realdon, Mariateresa Nardi, Cristina Fatigoni, Vincenza Gianfredi

**Affiliations:** 1 Nutritional Support Unit, Veneto Institute of Oncology IOV-IRCCS, Via Gattamelata 64, 35128 Padua, Italy; mariateresa.nardi@iov.veneto.it; 2 School of Medicine, Vita-Salute San Raffaele University, Via Olgettina, 60, 20132 Milan, Italy; marino.alessio@hsr.it (A.M.); gianfredi.vincenza@hsr.it (V.G.); 3 Digestive Endoscopy Unit, Veneto Institute of Oncology IOV-IRCCS, Via Gattamelata 64, 35128 Padua, Italy; 4 Department of Pharmaceutical Science, University of Perugia, Via del Giochetto 2, 06123 Perugia, Italy; cristina.fatigoni@unipg.it; 5 CAPHRI Care and Public Health Research Institute, Maastricht University, 6211 Maastricht, The Netherlands

**Keywords:** lifestyle, esophageal cancer, cancer prevention, esophageal adenocarcinoma

## Abstract

One of the most notable changes in the epidemiology of esophageal cancer (EC) is the rising incidence and prevalence of esophageal adenocarcinoma (EAC) in developed countries. The aim of this systematic review was to collect and summarize all the available evidence regarding lifestyle, diet, and EAC risk. We searched the PubMed and Scopus databases in January 2021 for studies providing information about lifestyle, diet, WCRF/AICR recommendations, and EAC risk; published in English; without a time filter. The Newcastle–Ottawa Scale was used to assess risk of bias. The results are stratified by risk factor. A total of 106 publications were included. Half of the case-control studies were judged as high quality, whilst practically all cohort studies were judged as high quality. Body mass index and waist circumference were associated with increased EAC risk. Physical activity did not appear to have a significant direct role in EAC risk. A diet rich in fruit, vegetables, and whole grains appeared to be more protective than a Western diet. Alcohol does not seem to be related to EAC, whereas smokers, particularly heavy smokers, have an increased risk of EAC. Prevention remains the best option to avert EAC. Comprehensible and easy to follow recommendations should be provided to all subjects. Protocol ID number: CRD-42021228762, no funds received.

## 1. Introduction

Esophageal cancer (EC), including squamous cell carcinoma (ESCC) and adenocarcinoma (EAC), is the sixth leading cause of cancer-related death (mortality rates 7.7 per 100,000) both in men and women worldwide, and the eighth most common cancer with approximately 604,100 new cases occurring in 2020 [1,2]. While the incidence of many types of cancer is expected to decrease over the next few decades, it is estimated that by 2040, esophageal cancer will increase by 63.5% [3]. One of the most notable changes in the epidemiology of esophageal cancer lies in the rising incidence and prevalence of EAC over recent years in developed countries (e.g., the United Kingdom, Australia, The Netherlands, and the USA) [1,4]. The higher incidence of EAC is recorded in males more than in females (the male-to-female incidence ratio is 4.4:1 for EAC) [5], and in Caucasians with a high socioeconomic status [1,6,7].

Given the rapid increase in the overall incidence rate and the variation in the change in rates among different geographic areas, it is likely that lifestyle and/or environmental factors, as well as genetic factors, play important roles in the development of esophageal adenocarcinoma [8]. In 2007, the World Cancer Research Fund and the American Institute for Cancer Research (WCRF/AICR) proposed a series of recommendations concerning the correct lifestyle approach to reduce the risk of cancer. In particular, these recommendations highlighted the importance of body weight control, physical activity, vegetable and fruit consumption, and limited intake of animal source foods, salty foods, alcohol, and nutritional supplements [9]. In their report, the WCRF/AICR indicated that the intake of fruit, non-starchy vegetables, β-carotene, and vitamins C and E was deemed “probably” protective against the risk of esophageal cancer, while the evidence linking fiber and folate intake to lower disease risk was described as “limited” [9]. The report also indicated that consumption of red meat and processed meat “probably” increases disease risk, while no food or nutrients were considered to have “convincing” evidence of an association with esophageal cancer. Unfortunately, the 2007 WCRF/AICR report did not discriminate between the two common histological types of esophageal cancers (squamous cell carcinoma and adenocarcinoma), even though these two malignancies have substantially different risk factors and etiology. In fact, the esophageal cancer section was updated in 2018 as part of the WCRF/AICR 2018 Continuous Update Project (CUP) Expert Report [10], differentiating between EAC and ESCC. However, in this report, body fat (marked by body mass index (BMI), waist circumference, and waist/hip ratio) was confirmed as a risk factor with convincing evidence for EAC. No protective factors with convincing or probable evidence are mentioned in the report. “Limited-suggestive” evidence in decreasing EAC risk is provided for vegetables and physical activity. On the contrary, no conclusive evidence was found regarding all other dietary and lifestyle factors, such as dietary fiber intake, and fruit and vegetable consumption [10]. Nevertheless, it should be considered that, due to the possible interaction between different foods and micronutrients, or the protective/causative role of other lifestyle habits, it is quite difficult to identify the real association between specific food components and EAC. It should also be taken into consideration that the 2018 CUP Expert Report was based on a 2016 literature review. Lastly, even if several systematic reviews and meta-analyses have been already available in the literature, all of them only focused on a single risk factor [11,12] or were restricted to some specific geographical region [13,14]. On the contrary, we believe that collecting and summarizing evidence on multiple risk factors is important in maintaining a holistic approach, which in turn is helpful in better exploring their overall role in real world. The aim of the present systematic review was primarily to collect and summarize all the available evidence concerning diet and other potential EAC risk factors and, secondly, to assess any potential new evidence.

## 2. Materials and Methods

### 2.1. Search Strategy

Two authors (D.N. and V.G.) independently performed a systematic search of published articles using the PubMed and Scopus databases up to January 2021. The search strategy was based on three parameters: esophageal adenocarcinoma, life styles, and study design. We used the following search terms combined with Boolean operators: (((((“Surveys and Questionnaires”[Mesh] OR “Cross-Sectional Studies”[Mesh]) OR “Cohort Studies”[Mesh]) OR “Case-Control Studies”[Mesh]) OR “Interview” [Publication Type] OR “population based” OR “food frequency questionnaire” OR FFQ)) AND ((((((((((“Fruit”[Mesh]) OR (“Vegetables”[Mesh] OR “Vegetable Products”[Mesh])) OR “Body Mass Index”[Mesh]) OR “Diet”[Mesh]) OR “Social Class”[Mesh]) OR “Tobacco Use”[Mesh]) OR “Smoking”[Mesh]) OR “Alcohol Drinking”[Mesh])) AND (“Adenocarcinoma Of Esophagus” [Supplementary Concept] OR “Esophageal Neoplasms”[Mesh:NoExp])). Both medical subject headings (MeSH) and free-text search terms were applied in order to maximize the citation search. In general, we preferred to use MeSH terms because they are more precise as compared with free text. Nevertheless, we only used free-text words for the concepts lacking an acceptable synonym in the Mesh database. However, it should be noted that behind MeSH terms, a lot of so called “entry terms” are connected and automatically included in the search. For this reason, we used a complex but synthetic search strategy. No time limitation was applied.

We also reviewed the reference lists from retrieved articles and those from previous review studies to identify additional relevant studies that may not have been identified by our database searches. Article screening for this systematic review was carried out manually and with the EndNote^®^9.0 (Clarivate^TM^, Philadelphia, PA, USA) software. The selection process was carried out independently by two authors (V.G. and D.N.), according to the Preferred Reporting Items for Systematic Reviews and Meta-Analysis (PRISMA) guideline [15]. The review protocol was registered on PROSPERO [16], the International Prospective Register of Systematic Reviews funded by the National Institute of Health Research (ID number CRD-42021228762 at https://www.crd.york.ac.uk/prospero/ (accessed on 5 January 2021) and formally registered on 5 February 2021).

### 2.2. Inclusion and Exclusion Criteria

The rationale for the selection of inclusion/exclusion criteria was based on the Population, Intervention, Control, Outcome, Study (PICOS) design framework [17]. This systematic review only includes articles in the English language, full length, carried out on adult humans (studies using animal or in vitro models were excluded), and those focusing on EAC. Epidemiological studies of any design (case-control, cross-sectional, or cohort studies evaluating the relationship between diet, BMI, lifestyle, and the risk of EAC) were included. Experimental animal models, genetic or immune-histochemical studies, and studies evaluating a combination of EAC and ESCC, or EAC and gastric adenocarcinoma, or EAC and gastric cardia adenocarcinoma were also excluded. Inclusion/exclusion criteria are presented in Table 1. Abstracts, case reports, letters, comments, reviews, and studies without appropriate data for extraction were excluded.

### 2.3. Data Extraction and Quality Evaluation

The data below was independently extracted from each study by two authors (D.N. and V.G.). Discrepancies and missing data were resolved by group discussion, and the opinion of another researcher (S.R.) was sought for further discussion in the case of any remaining discrepancies. As performed in previous published reviews [18], data were tabulated on a standardized and prepiloted data extraction form, and elaborated in Microsoft Excel^®^ 2013 for Windows (Microsoft Corporation, Redmond, WA, USA). The following data were extracted: the first author’s last name, the publication year, the study period, the country where the study was conducted, the study design (case-control, cohort, and cross-sectional), the number of cases and controls or the cohort size, the study aim, data extraction and, lastly, the results obtained in relation to EAC risk. The characteristics of included studies are presented in Tables which are stratified by study design. The quality of the included publications was assessed by two independent authors (A.M. and C.F.) using the Newcastle–Ottawa Scale (NOS) for observational studies and stratified by study design [19]. The NOS provides a checklist for case-control and cohort studies, but not for cross-sectional ones. For this reason, we used an adapted NOS for cohort studies in order to perform a quality assessment of cross-sectional studies, which is available in the literature [20]. As in previous systematic reviews, scores of 0–3, 4–6, and 7–9 were rated low, moderate, and high quality, respectively [21]. With regard to Item 7 of the NOS checklist for cohort studies, we determined that a 10 year follow-up period was acceptable for the occurrence of outcome of interest [22].

## 3. Results

### 3.1. Literature Search and Quality Evaluation

**Figure 1:**
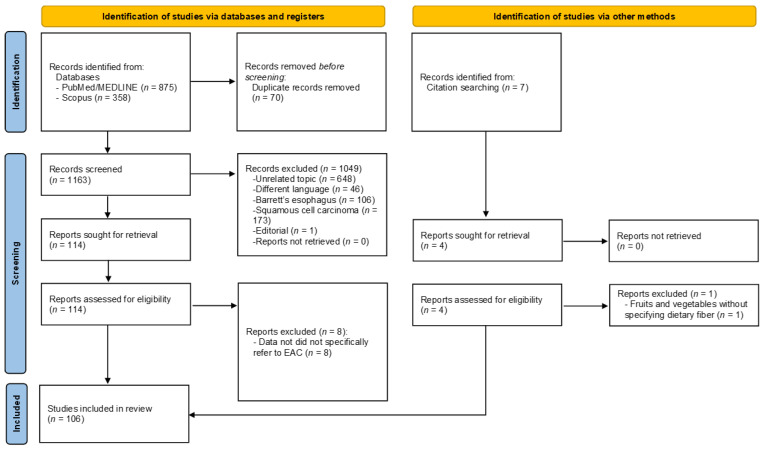
Flowchart depicting the studies’ selection processes (PRISMA flow diagram).

Our search strategy yielded 1240 articles. Among these, 1233 articles were found through an electronic literature search in the databases and 7 additional articles were found as references in the retrieved articles. These included 77 articles which were removed because they were duplicates, 46 articles removed because they were not in English, 75 articles were reviews, 648 articles were excluded because the topic was unrelated, 106 articles referred to Barrett’s esophagus, 173 articles were about ESCC, and 1 article was an editorial. A further 8 publications were excluded because data did not specifically refer to EAC, but rather a combination of EAC with squamous esophageal cancer or esophagogastric junction [23,24,25,26,27,28,29,30]. A final number of 106 publications were included in the systematic review. The selection process is depicted in Figure 1. The results are presented and grouped in accordance with the WCRF/AICR 2018 recommendations for cancer prevention [10]. The main characteristics of included studies are reported in Supplementary Table S1 for case-control studies (*n* = 66) [31,32,33,34,35,36,37,38,39,40,41,42,43,44,45,46,47,48,49,50,51,52,53,54,55,56,57,58,59,60,61,62,63,64,65,66,67,68,69,70,71,72,73,74,75,76,77,78,79,80,81,82,83,84,85,86,87,88,89,90,91,92,93,94,95,96] and Supplementary Table S2 for cohort (*n* = 39) and cross-sectional studies (*n* = 1) [97,98,99,100,101,102,103,104,105,106,107,108,109,110,111,112,113,114,115,116,117,118,119,120,121,122,123,124,125,126,127,128,129,130,131,132,133,134,135,136]. Table 2 reports a brief description of studies stratified by risk factors assessed, direction of the association, and quality evaluation.

According to the defined cut-points, more than half of the case-control studies were deemed high quality (*n* = 39/66, 59.1%); however, the rest contained a risk of bias. In fact, medium quality was assigned to 39.4% of the studies (*n* = 26/66) and low quality to 1.5% of the studies (*n* = 1/66). The main concerns were associated with the definition of controls (Item 4) not described in approximately 3 out of 4 studies (*n* =18/66), the non-response rate (Item 8) not described in 22.7% of the studies (*n* = 14/66), and a difference between cases and controls in half of the studies (33/66). Less than half the studies (*n* = 27/66, 40.9%) also had a satisfactory ascertainment of exposure (Item 6), with “interview not blinded to case-control status” being the most common outcome in those studies which did not collect a positive score. To the contrary, practically all cohort studies were rated as high quality (*n* = 38/39, 97.4%), while the remaining one study was rated as medium quality, achieving a score of 6 points. Information regarding lost to follow-up (Item 8) was not stated in 20.5% of the studies (*n* = 8/39), representing a potential risk of both information and selection bias. Another critical item was the follow-up length (Item 7), which was found to be insufficient for outcome to occur in approximately one-fourth of the studies (*n* = 10/39). With regard to the ascertainment of exposure (Item 3), 23% of the studies (*n* = 9/39) used a written self-reported questionnaire, which is at risk of potential recall and social desirability bias. Furthermore, 25.6% of the studies (*n* = 10/39) did not ascertain that EAC was absent at the beginning of the study. Lastly, a single cross-sectional study [103], deemed high quality (7 points), was included. Supplementary Table S3 reports the quality assessment of case-control studies and Supplementary Table S4 reports the quality assessment of cohort studies.

### 3.2. Anthropometric Measures

”Keep your weight within the healthy range and avoid weight gain in adult life” is the first recommendation given by the WCRF/AICR in 2018 [10]. A healthy weight range means a normal range of body mass index (BMI). BMI is defined by the World Health Organization (WHO) as the weight in kilograms divided by the square of the height in meters (kg/m^2^) [137]. Subjects are in a normal weight range when BMI falls within the range of 18.5–24.9, whereas they are defined as overweight if their BMI is in the range of 25.0–29.9, and obese if it is ≥30.0. With regard to weight control, several case-control and cross-sectional studies have demonstrated the association between a high BMI and the risk of EAC [38,39,40,42,53,57,63,75,80,81,86,88,93,95,103]. The studies included in our systematic review found a statistically significant higher risk of EAC in obese patients. Excess weight is a strong risk factor for EAC, with risk rising consistently as BMI increases. Risk appeared to be largely related to elevated BMI *per se* and not to weight gain or loss during adult life [39]. The association between BMI and EAC does not seem to be affected by symptomatic gastroesophageal reflux, although it appears to be synergic with BMI [40,57,63,75,81]. Visceral adipose tissue is another risk factor in the development of EAC and seems to be more important than obesity *per se* [65]. A larger abdominal diameter (with and without adjustment for BMI) was a risk factor for EAC, with a 10% increase in EAC risk for every centimeter of increased abdominal diameter in subjects with an abdominal diameter (anterior-posterior diameter) of >20 cm [40]. Drahos et al. [43] and Lin et al. [118] also investigated the relationship between other conditions often associated with obesity, such as metabolic syndrome (MetS), hypertension, hypercholesterolemia, and diabetes and the risk of EAC. In their study, the authors reported that obesity and hypertension were associated with EAC, but high cholesterol, type 2 diabetes, and MetS were not. Whereas only one study assessed the association between waist/hip ratio and risk of EAC finding no statistically significant association [32]. The cohort studies analyzed also all confirmed previous case-control study results [97,104,119,121,122,123,125,130,133], with the exception of three studies that failed to find a statistically significant association [110,116,131]. For EAC, each of the three BMI categories greater than normal significantly and progressively increased the risk of cancer. Being overweight in early adulthood and weight gain later in life were also associated with an increased risk of EAC [119,122]. Moreover, waist circumference, abdominal obesity [110], hip circumference [131], and waist/hip ratio (WHR) [121,126,130] were associated with a higher risk of EAC. In particular, Steffen et al. [131] analyzed data from the European Prospective Investigation into Cancer and Nutrition (EPIC) study and reported that abdominal, rather than general obesity, was a strong and robust risk factor for EAC. The authors also provided new evidence on the protective effect of gluteofemoral (subcutaneous) adipose tissue in EAC. These results were also obtained in a two-stage control function instrumental variable method of the Mendelian randomization analysis aimed at estimating the unbiased and unconfounded effect of BMI on the risk of EAC [86].

### 3.3. Physical Activity

”Be physically active as part of everyday life—walk more and sit less” is the second recommendation for cancer prevention proposed by the WCRF/AICR in 2018 [10]. Physical activity should be part of daily life. Indeed, the WCRF/AICR recommends at least 30 min of moderate-intensity activity per day, which can include occupational, transport, household, or leisure activities [9]. However, physical activity of a longer duration or greater intensity shows more beneficial health outcomes. Few studies investigating the association between physical activity and EAC are available. Data from the EPIC cohort show no association between EAC and any kind of physical activity (occupational, recreational, and household) at any level of intensity [111]. In 2013, Cook et al. [100] also reported no association between physical activity and EAC risk. The authors surprisingly showed an inverse association between sedentary behavior and EAC risk. In the multivariable Cox proportional hazards regression analysis, this inverse association was statistically significant in subjects who watched television for 3–4 h/day and 5–6 h/day. However, these results were not statistically significant after adjusting for multiple testing. A statistically significant association was instead found by Vigen et al. [89]. Indeed, in their population-based case-control study, a decreased risk of EAC was associated with an increase in the total activity index for the highest versus lowest quintile.

### 3.4. Dietary Patterns, Food Groups, and Beverages

The WCRF/AICR report suggests “Limit consumption of ‘fast foods’ and other processed foods high in fat, starches, or sugars” [10]. In this section, we analyzed the literature concerning the association between dietary patterns and EAC. In particular, we retrieved thirteen studies (twelve case-control studies [33,36,50,56,64,66,70,71,74,87,94,96], and one cohort study [117]) examining the relationship between dietary patterns and EAC. Mayne et al. [66] reported an association between plant-based foods (dietary fiber, carbohydrates, polyunsaturated fat and vegetable protein) dietary patterns and a reduction of EAC risk. Conversely, a higher intake of nutrients primarily provided by foods of animal origin (saturated fat, animal protein, and cholesterol) is associated with an increased risk. Chen et al. [36] showed a 3.6-fold higher risk of EAC for the “high in meat” pattern, a 2.9-fold higher risk for the “high in salty snacks” pattern, and a 2.6-fold higher risk for a diet “high in white bread”. In contrast, the daily consumption of fish, all vegetables, citrus fruit and juices, and dark bread were each associated with a 50% lower EAC risk. In one study, the authors classified patients into three dietary patterns [33]: (i) a “healthy diet” (prevalence of vegetables, tomatoes, fruit, fish, and poultry); (ii) a “Western diet” (prevalence of processed meat, red meat, sweets, high-fat dairy, and high-fat gravy); (iii) “alcohol drinker” (including French fries and alcoholic beverages such as beer and liquor). The results showed that the healthy diet pattern was, in general, associated with a moderately, not statistically significant reduced risk. In contrast, the Western diet pattern was associated with a modestly increased risk of EAC. Navarro Silvera et al. [70] reported a significantly increased risk of EAC in subjects with a dietary pattern characterized by a high intake of meat (particularly red meat), and a low intake of vegetables and fruit. In particular, when analyzing food subgroups, they found that red meat, high-fat dairy, and high-fat foods were associated with an increased risk of EAC, whereas raw vegetables were associated with a decreased risk. The same author confirmed these results in 2011, performing pattern analyses of dietary and lifestyle factors in relation to EAC risk [71]. In particular for the “fruit/vegetable” pattern, (mainly composed of deep yellow/orange and dark green and cruciferous vegetables, tomato products, citrus and noncitrus fruit) and the “meat/nitrite” pattern (mainly composed of nitrite, high-nitrite meats, and red meats). Significant inverse associations with the risk of EAC were found in the highest quartile of intake in the “fruit/vegetable pattern” as compared with the lowest quartile. However, the results regarding the “meat/nitrite” pattern were contrariwise. The authors reported a significant positive association for this pattern between EAC risk and the highest quartile of intake as compared with the lowest quartile. Ibiebele et al. [50] used three dietary patterns in their analysis: (i) “meat and fat”, (ii) “pasta and pizza”, (iii) “fruit and vegetables”. The authors found no association between EAC risk and the “pasta and pizza” and “fruit and vegetable” patterns. A statistically significant association was found between EAC risk and the “meat and fat” dietary pattern, characterized by a high intake of processed meat, high-fat potato, discretionary fat, red meat, high-fat dairy, poultry with skin on, white bread, sweet snacks, and fatty spreads, and a very low intake of fruits, vegetables, and fish. An increased risk of EAC was shown for this dietary pattern. After an analysis of the individual food that strongly contributes to the pattern, this association seems to be driven in part by high-fat dairy foods. In a large cohort study with almost 500,000 participants (women and men), Li et al. [117] examined the association of two diet quality indexes: the Healthy Eating Index-2005 (HEI-2005) [138] and the Alternate Mediterranean Diet (aMED) scores [139] and the risks of EAC. Higher HEI-2005 scores were associated with a significantly reduced risk of EAC, but not with aMED. Two recent study focusing on the relationship between the dietary inflammatory index (DII) [140] and the risk of EAC showed positive, statistically significant associations between DII scores and the risk of EAC in both studies [63,64]. A dietary pattern with a high proportion of carbohydrates showed a decreased risk of EAC [56,94], while a high proportion of fat increased the risk [56,74,87,96]. In detail, O’Doherty et al. [74] reported an increased risk of EAC in subjects with a high intake (53.8–54.8 g/day) of saturated fat, monounsaturated fat (41.2–41.4 g/day), polyunsaturated fat (24.8 and 27.7 g/day), and cholesterol (462.3–484.7 g/day). Opposite results on fats were obtained from a very large U.S. cohort study involving almost 500,000 men and women [120]. Following a multivariate analysis, this study found no association between total fat/fat subtypes and EAC risk. The authors only demonstrated a non-significantly possible protective role of polyunsaturated fat against EAC. Total protein in the diet does not seem to influence the risk of EAC [56] although Wolfgarten et al. [94] reported an increased risk associated with the intake of animal proteins ≥75 g per day.

#### 3.4.1. Foods of Plant Origin and Dietary Fiber

”Eat a diet rich in wholegrains, vegetables, fruit, and beans.” Fruit and vegetables, as well as legumes and whole grains, are the main sources of dietary fiber. According to the WCRF/AICR, fiber intake is likely to protect against several cancers [10]. To the best of our knowledge, the first study evaluating the association between vegetables/fruit consumption and EAC risk was Tzonou et al. in 1996 [87]. In this study, the consumption of vegetables and fruit, as well as the intake of crude fiber, were inversely associated with EAC [87]. Individuals eating ≥4 servings/day of fruit and vegetables had a 50–75% lower risk of EAC; however, no significant associations were found between any specific fruit or vegetable and EAC [80,84]. In 2007, Freedman et al. [106] found a significant association between Chenopodiaceae (spinach) intake and a low risk of EAC and a suggestive but not significant association between Cruciferae, Graminacae, and Leguminosae, and EAC risk reduction. The results from Freedman et al. were confirmed by Steevens et al. [129] who showed a significant inverse association between raw leafy vegetable intake and EAC risk, while the consumption of Brassica vegetables was associated with a slightly decreased risk of EAC per 25 g/day increments, as were tomatoes and onions. In the EPIC study, the authors also showed a negative, although not significant, association between vegetable intake leafy vegetable intake and EAC risk [108]. A possible protective role of fruit in reducing EAC risk has been confirmed in several studies [32,38,129], with a linear relation [38], and in particular for citrus fruits [129]. However, three studies [106,108,129] found a non-significant inverse association between fruit intake and EAC risk. Moreover, four case-control studies found a protective effect of fiber intake against EAC [35,58,66,96]. In particular, Wu et al. found that a high total fiber intake (>15 g/day) was associated with a statistically significant reduction of EAC risk, and this inverse association appeared to have a stronger effect if the fiber source originated from fruit and vegetables instead of cereals. Data confirming the strong association between fiber intake and EAC risk reduction were reported by Lahmann et al. [58]. The authors analyzed data from a large Australian population-based case-control study and showed a statistically significant inverse association between fiber intake and the risk of EAC, with a risk reduction of 28–37% per 10 g/day.

#### 3.4.2. Animal Products

With regard to animal products, the WCRF/AICR suggests “limit consumption of red and processed meat” [10]. However, results on the association between meat consumption and EAC risk are not unanimous. Ward et al. [90] found a significantly higher risk with increasing red meat intake (primarily processed meats and beef). In this case-control study, a high intake of processed meats was associated with an elevated risk of EAC, whereas a high beef intake was not. The highest frequency of intake of red meat (>8 times/week) and gravy (>4 times/week) was associated with about a 2-fold increased risk. As retrieved also by Ward et al. [92]. Through the EPIC study, Gonzalez et al. [109] found a not statistically significant association between EAC risk and total meat and processed meat intake, and a positive association between poultry intake and EAC. In another EPIC cohort study, Jakszyn et al. found a significant association between a higher intake of processed red meat and an increased risk of EAC when the third tertile of intake was compared with the lowest tertile. However, the association did not remain significant when intake was considered as a continuous variable [112]. Lastly, in this study, no significant association was found between a higher intake of white meat or unprocessed meat and the risk of EAC [112]. No statistically significant association between red and processed meat and with meat and the risk of EAC was also detected in another cohort study [101]. Wu et al. (2007) found no association in their case-control study between total meat consumption and EAC risk [96]. However, the risk tended to increase for red and processed meat, but it was not statistically significant. In the Netherlands Cohort Study (NLCS) involving 120,852 subjects (58,279 men and 62,573 women), red and processed meat intake were not associated with EAC risk [114]. Another study showed no consistent association between total red meat (fresh and processed) intake and EAC risk, but patients in the highest level of intake (69.8–72.8 g/day) of fresh red meat had a higher risk of EAC as compared with the controls [74]. An increase in EAC risk was seen in patients in the fourth quartile of corned beef/luncheon meat and in the fourth quartiles of beef and lamb intake. Those in the second quartile for sausage were also at increased risk of EAC. In accordance with the majority of studies mentioned above, Navarro et al. reported, in 2014, that consuming more than half a serving of red meat per day was associated with a high risk of EAC [72]. As regards the consumption of dairy products, this showed a positive association with EAC risk, although it was not statistically significant [68].

#### 3.4.3. Consumption of Nonalcoholic Beverages

The WCRF/AICR report suggests “limit consumption of sugar sweetened drinks” [10]. According to a case-control study, the intake of sweetened beverages was associated with an increased risk of EAC [59]. To the contrary, one cohort study [124] and two case-control studies show no association between carbonated soft drink consumption and risk of EAC. In particular, Lagergren et al. conducted a study among a Swedish population [55]. The study enrolled 189 patients and 820 controls. They conducted an interview to investigate the food and beverage consumed 20 years earlier. Mayne et al. [67] conducted another case-control study, in 2006, having a similar study design and sample size (282 cases and 687 controls). They conducted a survey evaluating the beverages consumed 5 years earlier and they found an inverse association between diet carbonated soft drink consumption and the risk of EAC, whereas no association was found with regular carbonated soft drink.

Contrasting results also emerged for hot beverages (>65 °C), tea, and coffee consumption, as confirmed by limited nonconclusive evidence reported by the WCRF/AICR [10]. In particular, we found two cohort [124,135] and one case-control studies [48]. The case-control study [48] found a statistically significant association between hot or very hot beverages and EAC, whereas coffee consumption showed a dose-response increased risk that was not statistically significant. Similar results were also found in the two cohort studies [124,135], particularly the most recent EPIC cohort study [135] which evaluated the possible relationship between coffee and tea consumption and the risk of EAC. They found no statistically significant association between tea and coffee consumption and esophageal cancer even after exploring the two histological types (EAC and squamous). The dose-response increased risk was confirmed for coffee consumption, even if not significant, while an inverse but not significant association between tea consumption (in particular black tea) and EAC was found. There was also no statistically significant interaction between tea/coffee intake and baseline alcohol intake or BMI.

### 3.5. Vitamins, Minerals, and Other Nutrients

The WCRF/AICR report suggests “do not use supplements for cancer prevention, aim to meet nutritional needs through diet alone” [10]. A recent large U.S. population-based study reported a risk reduction of around 57% for EAC incidence due to dietary anthocyanidins, although a modest 13% decreased risk of mortality among EAC patients was observed [79]. A not statistically significant association between a dietary intake of flavonoids and overall EAC risk was reported in the EPIC cohort. Nevertheless, a significant inverse association between total dietary flavonoids, flavonols, flavanols, and flavan-3-ol monomers and EAC risk was only shown in current smokers [132]. Two Swedish nationwide and population-based case-control studies explored the association between a dietary intake of lignans, quercetin, and resveratrol and EAC risk. In the first study, the authors found a non-significantly reduced risk [62], conversely, the second, more recent study, showed a significant association between the intake of lignans, quercetin, and resveratrol, and a decreased risk of EAC [61].

#### 3.5.1. Vitamin C

As regards vitamin C, the WCRF/AICR report [10] found limited nonconclusive evidence. However, we retrieved five case-control studies [34,66,69,83,87] which all reported the protective role of a dietary intake of vitamin C. The first findings by Tzonou et al. [87], in 1996, emerged from a hospital-based case-control study that showed an inverse association between vitamin C and EAC. These results were confirmed in nationwide population-based case-control studies from Sweden [83] and Germany [34]. Consistently, a multicenter case-control study [66], which particularly focused on vitamin C plant food, and an all-Ireland population-based case-control study [69] reported that patients with a lower risk of EAC are those with the highest intake of vitamin C. In particular, the latter found a significant reduction in risk for a vitamin C intake higher than 168 mg/day. A further reduction in risk in current smokers was found with the highest intake of vitamin C.

#### 3.5.2. Vitamin E

The WCRF/AICR report [10] found limited nonconclusive evidence on vitamin E. In our review, the association between Vitamin E and EAC risk was investigated in four case-control studies [34,51,69,83] and one cohort study [99]. A significant risk reduction in the occurrence of esophageal tumor with an increased intake of vitamin E was reported by Bollshweiler et al. [34] and confirmed by Ibiebele et al. [51] in 2013. In particular, Ibebele et al. described a statistically significant decreased risk of EAC with a high intake (median daily intake 9.6 mg) of vitamin E from food sources and from a combination of food and supplements. In contrast with these findings, two case-control studies [69,83] and one large U.S. prospective cohort study [99] involving 492,559 participants reported no association between α-tocopherol, and γ-tocopherol intake and EAC risk.

#### 3.5.3. Vitamin A and Carotenoids

The WCRF/AICR report [10] found limited nonconclusive evidence with regard to vitamin A and carotenoids. In our review, the association between EAC and the intake of vitamin A and carotenoid (total carotenoids, β-carotene, and β-cryptoxanthin) showed an inverse association in four [35,66,83,87] of the five [35,66,69,83,87] studies analyzed. The majority of the studies reported a 40–50% risk reduction with evidence of a dose-response effect at high intake. In contrast with previous results, Murphy et al. [69] reported, in 2010, that an all-Ireland population-based case-control study resulted in no association between total carotenoid intake and EAC risk.

#### 3.5.4. B Vitamins

The WCRF/AICR report [10] found limited nonconclusive evidence for B vitamins and, specifically, pyridoxine (B6), folate, thiamin (B1), and riboflavin (B2). We found four studies (three case-control and one cohort) that investigated folate intake and EAC risk [35,66,82,134]. The three case-control studies reported an inverse significant association between folate intake and EAC, with an approximate 50% risk reduction as a result of high folate consumption [35,66,82]. Although previous studies found a significant inverse association between dietary folate intake and EAC, recent data from a large U.S. cohort of 492,292 persons showed that higher folate intake was not associated with EAC risk. Moreover, no association was observed between total folate intake (diet + supplement) and EAC risk [134]. Vitamin B6 showed an inverse association with EAC risk in one [66] out of two studies [66,134]. Opposite results were found for vitamin B12 [82], which showed a significant increased risk of EAC. In contrast with these results, in a recent large U.S. cohort including almost 500,000 persons, Xiao, et al. [134] reported no association between the intake of vitamins B6 and B12 and EAC risk.

#### 3.5.5. Vitamin D and Calcium

The associations between EAC, and vitamin D and calcium are currently understudied. Indeed, specifically for calcium, the WCRF/AICR report [10] found limited nonconclusive evidence. An all-Ireland population-based case-control study evaluated the role of vitamin D and calcium in EAC risk [68]. The authors observed a significant direct association between subjects with the highest vitamin D intake (≥3.0–9.7 μg/day) as compared with those at the lowest level of intake (<2.05 μg/day). Dietary calcium does not seem to be associated with EAC risk [68].

#### 3.5.6. Iron

With respect to the role of iron in EAC, the WCRF/AICR report [10] found limited nonconclusive evidence. In 2001, Wolfgarten et al. [94] reported that a daily consumption of more than 18 mg of total iron (heme and nonheme iron) was inversely correlated with EAC. Moreover, on the one hand, two population-based case-control studies, conducted in Ireland and in the U.S., showed a positive direct association between heme iron intake and EAC risk [73,92]. Consistent results were also found in the EPIC cohort study, according to which a higher intake of heme iron was significantly associated with a higher hazard of EAC [112]. A suggestive positive association was also found in a U.S. cohort study [101] whereas, in a Netherland cohort study [115], researchers found no apparent associations between heme iron intake and EAC. On the other hand, nonheme iron intake showed a statistically inverse association with EAC. This inverse association was confirmed per 10 mg/day increments [73]. However, contrary to the results from Ward et al. [91], three studies reported no association between EAC and nitrate, nitrite [92,101,115] and N-nitroso compounds (NOC), whose endogenous formation in the lower gastrointestinal tract in humans is also influenced by heme iron [115].

#### 3.5.7. Other Compounds

No significant association was observed for higher intakes of methionine [134]. The intake of magnesium (Mg) and EAC risk is controversial. In a German case-control study, the authors reported an inverse correlation between a daily Mg intake of more than 500 mg and EAC risk [94]. A recent all-Ireland population case-control study [41] with 226 EAC cases showed no significant association between Mg intake and EAC risk. In 2002, Chen et al. [35] described an inverse association with a risk of EAC for dietary intakes of Zinc, whereas no association between EAC risk and the intake of selenium, copper, and zinc was found in a further study [69]. Conversely, an inverse association between selenium status and risk of EAC was shown in women, never smokers, and in low antioxidant consumers [128].

#### 3.5.8. Dietary Supplements

Concerning micronutrient intake from fortified foods and supplements and the relationship with EAC risk, Bollschweiler et al. [54] showed a significant risk reduction for EAC with increased folic acid intake. No associations were found between higher doses of a vitamin E supplement and risk of EAC [99]. A recent large U.S. cohort study with almost 500,000 subjects showed no significant association between multivitamin use and EAC risk [102]. As for an individual vitamin or mineral supplement intake, the authors found an inverse association between iron supplement use and the risk of EAC. A direct association emerged between EAC risk and the use of a calcium supplement. No further associations were found with the intake of any other individual vitamin or mineral supplements (zinc, selenium, folic acid, vitamin A, β-carotene, vitamin C, and vitamin E) and EAC risk [102]. A protective role of vitamin C and multivitamin supplements was reported in only one study [87]. In a large population-based case-control study, conducted in Ireland in 2010, the overall antioxidant index obtained by the combined intake of vitamin C, vitamin E, total carotenoids, and selenium was associated with a reduced risk of EAC [69]. These findings were confirmed, in 2013, through an Australian population-based case-control study, which demonstrated a decreased risk of EAC in subjects with a high score on the antioxidant index from food sources [51].

### 3.6. Cooking Process and Chemical Modification during Cooking

In 2007, the WCRF/AICR’s guidance regarding “preservation, processing, and preparation: limit consumption of salt, avoid moldy cereals (grains) or pulses (legumes)” was included as one of the ten cancer prevention recommendations [9]. Although this recommendation was not mentioned as one of the ten final recommendations in the last edition of the WCRF/AICR expert report [10], the importance of preserving, processing, and preparing food is mentioned in the report. We did not retrieve articles specifically analyzing salt consumption or exposure to aflatoxins and EAC, yet this recommendation is also in accordance with the “Continuous Update Project: Diet, Nutrition, Physical Activity, and Oesophageal Cancer” [10]. This paragraph presents results concerning modifications due to food preparation techniques and cooking processes. In the first population-based case-control study, frying, broiling, and grilling were the most commonly reported cooking techniques for beef [90]. Frying or broiling was not associated with risk of EAC. Grilling/barbecuing was associated with a 50% not significantly elevated risk of EAC [90]. The ORs for barbecuing were 3.1. Broiling/frying pork or chicken was not associated with the risk of EAC. Even doneness preference was not strongly or monotonically associated with EAC risk [90]. Among the chemical compounds formed during the cooking process, acrylamide has been shown to be one that potentially increases the risk of developing cancer for consumers in all age groups [141]. Acrylamide forms naturally in starchy food products subjected to high-temperature cooking (>120 °C) such as frying, baking, and roasting. In our review, only one retrieved case-control study [60] showed that the adjusted risk of EAC was higher, but not significant, among participants in the highest quartile of acrylamide exposure (≥44.08 μg/day) as compared with the lowest quartile (<27.27 μg/day). The risk was higher among overweight or obese people with a high intake. However, no dose-response association was observed [60].

Heterocyclic amines (HCAs) are the other chemicals formed during the cooking process which seem to increase cancer risk in humans. These compounds are mainly formed in meat and fish cooked at high temperatures. HCAs are formed in greater quantities when meats are overcooked or blackened [142]. No conclusive results were obtained in our review for HCAs and the risk of EAC. In a 2003 Swedish nationwide, population-based case-control study with 185 EAC patients, Terry et al. [85] did not find any association between a dietary intake of HCAs and EAC risk. In 2011, Cross et al. [101] found a positive association between HCA intake and EAC. In particular, a borderline statistically significant increased risk of EAC was found for those with the highest intake (25 ng/1000 Kcals) of MeIQx (2-amino-3, 8-dimethylimidazo [4,5-f] quinoxaline) and the highest intake (127.3 ng/1000 Kcals) of PhIP (pyridine).

### 3.7. Alcohol

Alcohol and alcoholic beverages are carcinogenic substances (group 1) for humans, as the International Agency for Research on Cancer declared in 2009 [143]. “Limit alcoholic drinks” is the WCRF recommendation [10]. Although the link between alcohol intake and many cancers are well established, the association between EAC and alcohol consumption is not completely clear. In fact, it is particularly hard to distinguish the possible effect due to dosage, duration, frequency of alcohol intake, and possible patient behavioral changes after diagnosis. In our review, we found 11 case-control studies [31,37,46,47,48,49,54,63,77,88,95], 7 cohort studies [98,105,107,110,113,127,133], and 1 cross-sectional study [103] evaluating the association between alcohol intake and the risk of EAC. From among our 11 case-control studies, three studies found a statistically significant association between alcohol consumption and risk of EAC. In particular, the studies conducted by Garidou et al. [48] and Chen et al. [37] showed that consuming more than five drinks/day was a risk factor for EAC. Their results showed ORs that are 5 to 24 times higher in heavy drinkers (daily alcohol consumption >30 mL/day) independent of the duration assumed [37], whereas EAC did not appear to be strongly associated with alcohol consumption in two case-control studies [47,49] and two cohort studies [105,107]. The first author to find no association between alcohol intake and EAC was Lagergren et al. [54]. In this study, never users of alcohol had a higher risk of EAC as compared with ever users. Beer and wine consumption was not associated with a risk of EAC, but users of hard liquor ran a low risk. This negative association, however, was not dose dependent [54]. One year later, Wu et al. [95] also confirmed that excessive use of alcohol was not associated with the risk of esophageal adenocarcinoma, as did the prospective nested case-control study conducted by Lindblad et al. in 2005 [63]. Lastly, Pandeya et al. [77] found no evidence of an alcohol dose effect for EAC and no evidence of any association (linear or nonlinear) between average lifetime beer intake and risks of EAC. Inversely, the risks of EAC were reduced significantly among those with very low intakes of sherry or liqueur (<10 g/wk) and a low to moderate intake (<90 g/wk) of wine. The potential protective role of wine was previously found by Gammon et al. in 1997 [46]. However, these results were not confirmed in a prospective hospital-based case-control study [88]. Another author confirmed the absence of association in a 2009 large population-based case-control study in Ireland, while also evaluating the historical (at age 21 and 5 years before the interview date) total alcohol consumption [31].

Among the six cohort studies retrieved, Ji, J. et al.[113] showed an increased risk of EAC in subjects with a heavy alcohol intake; in contrast, Steevens, J. et al.[127] found no association between alcohol intake (≥30 g/day) and the risk of EAC. Similar results were also found by Allen et al. [98] in their UK cohort study, according to which no alcohol intake threshold assessed (≤2, 3–6, 7–14, and ≥15 drinks/week) was statistically significantly associated with the risk of EAC. Conversely, two articles [107,110] did not find a statistical association, whereas Yates et al. [133] found an inverse association between alcohol intake and EAC risk.

### 3.8. Smoking

In addition to the ten cancer prevention recommendations, the WCRF/AICR report suggests “not smoking and avoiding other exposure to tobacco” [10]. In our review, all the retrieved studies concur in defining smoking as an important risk factor for EAC [32,37,44,45,46,47,48,63,76,78,80,88,93,95,103,105,110,127,136], except in three studies [49,54,107] where the association between tobacco smoking and the risk of EAC was increased but not significant. In particular, Lagergren et al. found a weak or absent association among persons who had smoked more than 20 cigarettes daily for more than 35 years as compared with never smokers. Conversely, the ORs ranged between two and four considering all the above-mentioned studies that found a detrimental effect of smoking. No gradients in risk were seen for the smoking duration; however, this data was disconfirmed by Chen et al. [37]. Focusing on the relationship between smoking and two other confirmed risk factors for EAC, obesity and GERD, Whiteman et al. [93] and Pandeya et al. [78] found that obesity was not associated with increased risk if considering smoking duration, but significant in heavy smokers as compared with modest smokers [93], whereas the combination of obesity and GERD was associated with a 60% higher risk of EAC [78].

After an in-depth analysis on the type of smoke, Lagergren et al. [54] found a higher risk of EAC among frequent pipe smokers as compared with never smokers. Cigar smoking was not associated with the risk. There was a declining risk with time since cessation of smoking. Snuff users had a not significant increased risk of EAC as compared with never users. Those using 15–35 quids per week showed a statistically significant 2-fold increase in risk as compared with never users.

We found controversial results with regard to the potential protective effect of smoking cessation. In a study by Wu et al. [95], the risk of EAC remained significantly elevated among former smokers who had quit smoking 10–19 years earlier. Similar results were also found by Freedman et al. in a prospective study considering both current and former smokers [105]. In their case-control study, Pandeya et al. found that the ”time since quitting” was independently associated with an approximate 15% reduction in risk per decade [76], as also found by Whiteman et al. [93]. A significant 2-fold increase in risk was found among previous smokers and among persons who had been smoking for more than 35 years [54]. Gao et al. and Lindblad et al. also estimated the risk of EAC according to gender. Gao et al. [47] found a 60% higher risk for women than for lifelong nonsmokers. Lindblad et al. [63] did not find significant differences between men and women. Passive smoking is another important risk factor for different types of diseases. Duan et al. [44] evaluated the risk of EAC in a study involving nonsmokers exposed to passive smoking during childhood. These subjects did not show a higher level of EAC risk than those with no exposure to passive smoking. Exposure to passive smoking during adulthood was associated with a raised risk of EAC; an increased risk of EAC was also observed in adults who were exposed to passive smoking for a long time [44].

Our investigation also included two cohort studies that had analyzed tobacco use and EAC risk [127,136]. Both concurred in considering smoking as a real risk factor. Current and former smokers had an increased risk of EAC as compared with never smokers. Smokers (current and former) who used cigarettes had a higher risk than those who only smoke cigars or a pipe [136]. In Steevens et al., the association between the frequency of cigarette smoking and the risk of EAC was statistically significant, with a statistical significance for 10 and 20 years after smoking cessation [127]. The risk of EAC did not increase for moist snuff users [136].

### 3.9. Interaction between Smoking and Alcohol

This section evaluates the possible synergic effect of tobacco and alcohol on EAC. We found a total of five case-control studies and three of the studies did not find a synergic effect for any of the smoking strata [37,49,77]. Inversely, in a study by Gao et al.[47], the risk tended to rise with increasing alcohol intake within each smoking category, except for nonsmokers, and with increasing smoking levels within each alcohol category, including nondrinkers. The combined effect of smoking and drinking alcohol was pronounced among men; the OR for those who smoked more than 1 pack per day and drank more than 750 g of ethanol per week was 12.0 (95% CI 6.60–22.10). Lagergren et al. [54] also found a smaller but still significant increased risk.

### 3.10. Socioeconomic Factors and EAC Risk

In 2006, Veugelers et al. [88] did not find a statistically significant association between educational level and EAC risk; to the contrary, in 1997, Gammon et al. [46] found that a high educational level had a protective effect; the same also held true for income level. In 2005, Jansson et al. [52] found a statistical association between socioeconomic status and EAC in a crude model, which was no longer significant after adjustment for BMI, reflux, and smoking habits. They also found the same results for educational level and for living in urban instead of rural areas. Interesting, however, is the statistically significant association between EAC and the number of cohabitants. Single people had twice the increased risk of EAC as compared with those who had a partner [52].

## 4. Discussion

To the best of our knowledge, this is the first systematic review providing a comprehensive overview of different types of lifestyles related to EAC risk alone. We carefully excluded all studies analyzing a combination of EAC and ESCC, and EAC combined with gastric cardia adenocarcinoma. In brief, our review found that anthropometric measurements such as high body weight, abnormal BMI (overweight and obesity) [38,39,40,42,53,86,95,97,103,104,119,121,122,125,130,133], and high waist circumference [40,110,118,121,126,130,131], along with a Western dietary pattern [33,36,50], high score in dietary inflammation [63,64] and a dietary pattern mainly animal bases [66,71], and smoking [32,45,46,63,95,103,105,110,127,136], increase the risk of EAC, especially focusing on studies with the highest quality (NOS ≥ 7). Conversely, high hip circumference [131], along with healthy dietary pattern [117] mainly based on plant foods [66,70,71] (fruits [32,38], vegetables [84,87,129], and dietary fibers [66,87,96]) together with foods rich in polyphenols [61,132], vitamins (C [66,69,83,87], carotenoids [66,83,87]) and folates [66,82] reduce the risk of EAC, if only studies with high quality are considered (NOS ≥ 7). Lastly, alcohol consumption is associated with controversial results or no effect on EAC [31,45,55,63,95,105,107,127], when only studies with high quality are considered (NOS ≥ 7). In other words, maintain an adequate body weight, reduce animal-based food and processed foods intake, increase plant food consumption, and avoid smoking and excessive alcohol consumption, should be the crucial points on which to focus efforts for esophageal adenocarcinoma prevention. Additionally, an important factor is the socioeconomic status [46,52], which is strictly related to diet and environmental exposure. In fact, descriptive epidemiology suggests a positive trend in EAC incidence, particularly in high-income countries. Certain areas of research, such as salty food and EAC specifically, were also not explored or the results of studies are not conclusive, as in the case of alcohol intake and EAC. Data regarding the association between carbonated drinks and the risk of EAC, the dietary intake of vitamins such as vitamin D and calcium, as well as cooking processes and chemical modifications during cooking were scant and inconsistent and future investigations should mainly focus on these aspects.

More in details, according to our results, weight control is an important factor in the prevention of EAC. Indeed, BMI is unanimously defined as an independent risk factor for EAC that does not appear to be associated with GERD. A higher-than-normal BMI (≥25.0 kg/m^2^) is significantly and progressively associated with an increased risk of EAC, as is body weight and waist circumference alone. These results, confirmed in all analyzed studies independently of study design [14,144,145], highlight the importance of maintaining anthropometric parameters within normal values in both males and females. Moreover, the higher the BMI, the higher the risk of EAC [145]. Furthermore, BMI > 30 kg/m^2^ was most strongly associated with early-onset (<50 y) EAC, and with significant differences across age groups. The magnitude of the association was higher in early-onset EAC than in later-onset patients. ORs for the other age categories ranged between 2.6 and 2.8 [146]. We can conclude that the elevated risk related to a high BMI probably represents a causal effect.

Even though the beneficial effect of physical activity is well known, EAC does not appear to be positively affected by physical activity. Indeed, our review only resulted in one population-based case-control study showing an inverse association between the total amount of physical activity and the risk of EAC [89].

In contrast, nutrition appeared to play a crucial role in EAC prevention. Although it is not easy to precisely assess dietary intake and to homogenously define dietary patterns, our results, in accordance with previous meta-analyses of observational studies [147], suggest that the “Western dietary pattern”—typically poor in vegetables, legumes, and whole grains and high in red meat and especially processed meat—is associated with an increased risk of EAC. However, there appears to be conflicting results in studies that focused on meat consumption. Indeed, it is not unanimously affirmed, even if most of the included studies found an increased risk of EAC in subjects with a high consumption of meat (particularly red and processed meat). These contrasting results could be due to the intrinsic limitation of single studies where the total sample size is generally limited. Subsequent meta-analyses consistently found an increased risk of EAC for a high intake of meat, considering both total meat intake [148] and red and processed meat [149,150] in both case-control and cohort studies. Additionally, the meta-analysis conducted by Huang et al. [150] assessed the risk of red and processed meat separately and, also in this case, the results confirmed an increased risk of EAC for the highest intake as compared with the lowest intake, which is slightly higher for processed meat consumption as opposed to red meat consumption. High risk was also confirmed in the dose-response analysis, which showed a higher risk per 100 g/day of red meat intake and per 50 g/day of processed meat intake [150]. On analyzing other animal products, we found no association between fish and EAC risk, as described in Han et al. [151] and Zhu et al. [148].

By contrast, the results of studies included in our systematic review suggest that a “healthy dietary pattern” rich in fruit, vegetables, and whole grains has a protective role, as opposed to a diet rich in animal fat, meat, processed meat, fried, or salty foods. On the basis of this growing evidence, we can hypothesize that a “healthy dietary pattern” is characterized by a high dietary intake of fiber. In actual fact, fiber intake has a biologically plausible explanation in cancer prevention [21,152,153], including EAC prevention through the binding of possible carcinogens, removing damaged cells from the esophageal epithelium [154,155,156], and positively modifying esophageal microbiota [157]. Moreover, in vitro studies also demonstrated a possible direct role of fiber in promoting apoptosis and inhibiting cell growth, even among esophageal adenocarcinoma cells (cell lines) [158]. Furthermore, fiber in food is associated with several bioactive compounds, such as polyphenols, that could have positive effects on modulating inflammation and reducing proinflammatory cytokine interleukin-6 concentrations [159].

In humans, it is associated with reduced gastroesophageal reflux symptoms, glycemic response, gastric emptying, and overall calorie intake helping in weight control [154,160,161]. Studies included in our review demonstrated the protective role of foods of plant origin (fruit and vegetables), in line with previous meta-analyses [86,162] which estimated a risk reduction of 24% and 27%, respectively, for the highest intake of vegetables and fruit, and approximately 30% for a combination of the two. The protective role of fruit and vegetables is probably due not only to the fiber amount, but also to the vitamin and antioxidant compound intake [162,163,164]. Although the meta-analysis showed the protective effect of fiber intake, there was a high statistical heterogeneity. The high heterogeneity is probably due to the unquantified fiber intake in the majority of included studies, such as the recall bias intrinsic in primary studies and the inclusion of case-control studies instead of cohort studies (which are known to be superior to case-control). Indeed, fruit and vegetables contain an important amount of both vitamins and antioxidants, which appear to be much more effective than supplements. Our results are in line with a previous meta-analysis which reported a 50% lower risk of EAC in subjects with a high intake of dietary vitamin C, with a dose effect at high intake [165]. With regard to vitamin E, a meta-analysis found a slight but non-significant reduction in EAC risk [165]. However, the results of our systematic review highlight that dietary vitamin intake is much more effective than vitamin supplementation, with the exception of iron and folic acid. This important phenomenon is probably due to the possible interactions and synergistic combinations of the several bioactive compounds contained in vegetables instead of “pills”, which still remain extremely useful in the case of clearly diagnosed deficiencies.

As described in previous studies, cooking methods may be related to an increased risk of upper gastrointestinal tract cancers [85,166,167,168]. According to WCRF/AICR recommendations, cooking methods that typically involve high temperatures (such as grilling, baking, and frying) can lead to a variety of potential carcinogens [169]. Baked or fried potatoes, bread (crisp or soft), cookies, and coffee can particularly contribute to an increased dietary acrylamide intake. Cooking meat at high temperatures can give rise to the formation of PAHs and HCAs [170,171]. These compounds have been suggested to increase the total risk of esophageal cancer [85,172,173]. Our review revealed a positive, but not statistically significant, trend between the daily intake of acrylamide and EAC risk, and mostly in obese patients. HCAs may also play a role in increasing the risk of EAC, but the positive trend that we found was not statistically significant.

With reference to nonalcoholic beverage consumption, we found contrasting results when both (carbonated) soft drinks and the hot beverages, coffee, and tea, were considered. We do not have a clear idea of the reasons behind this; however, it could be due to the intrinsic limitations of the studies since these are based on surveys and can be affected by several biases including a social desirability bias, recall bias, or dietary assessment performed after diagnosis, and which may not reflect intake in the distant past. When it comes to coffee and tea, the contrasting results can also be explained given that these drinks are rich in flavanols and flavanols have been demonstrated to have anticarcinogenic effects [174,175]. Caffeine is a well-known factor capable of reducing esophageal sphincter contraction (a cause of reflux) [176].

Tobacco and alcohol are two of the main risk factors causing several types of cancer. We analyzed the link between alcohol and EAC and tobacco and EAC separately, and the interaction between alcohol and smoking on EAC risk. Although alcohol consumption is linked to cancer of the oral cavity, pharynx, esophagus, liver, colorectal, and breast in women, it does not seem to be related to EAC [177,178,179]. Contrasting results were also found when considering alcoholic beverages. Even if some meta-analyses found a significant association between a lower alcohol intake and EAC [180,181], no dose-response effect was found. Moreover, we failed to find clear evidence that any particular type of beverage (beer, liquor, or wine) was especially associated with an increased or decreased cancer risk, as also confirmed by the meta-analysis by Tramacere et al. [182]. With regard to smoking habits, data in our review suggest that smokers, particularly heavy smokers, are at high risk of EAC. This evidence is in line with two pooled analyses which also confirmed a consistent dose-response association [180,183]. Risk also seems to persist in former smokers, as confirmed in a pooled analysis [183] and in a meta-analysis of 13 studies (9 case-control and 4 cohort), where the risk for former smokers was lower as compared with current smokers, but was still present after smoking cessation.

### Strengths and Limitations

Even though this systematic review offers an extensive overview of the potential relationships between EAC and several lifestyles, there are some limitations with regard to both the included studies and the review per se. We only included observational epidemiological studies that assessed the relationships between certain human behaviors (smoking habits, nutrition status, food habits, etc.) and health outcomes, in particular EAC risk [184]. In the majority of included studies, food intake was assessed through an FFQ evaluating dietary habits before cancer diagnosis. This aspect needs to be considered because of possible recall bias. Recall bias is a systematic error resulting from the imperfect recall of exposure, particularly true in retrospective studies [185]. Nevertheless, the FFQ appears to be one of the best methods to measure historical exposures. In the majority of studies, the FFQ was administered by an interviewer, which increases the quality and accuracy of data gathered. Our review’s limitations include the language filter, since we only included articles published in English, which could introduce potential bias. Excluding languages other than English may introduce a language bias and lead to the exclusion of some relevant studies [186]. We performed a structured computer search on two databases, as recommended by international guidelines (Supplementary Tables S5 and S6). Taking into consideration the type and the nature of the search question, we believe that it covered the majority of relevant potential sources of evidence, especially because our study aimed to offer an updated summary of the evidence-based literature available to improve the statements’ consistencies [187]. Moreover, the broad inclusion criteria allowed us to include different areas of interaction between potential risk factors and EAC [187]. Lastly, the quality of included studies was generally high. More specifically, the vast majority of cohort studies obtained the highest score as compared with case-control studies. Consequently, it can be concluded that our results are reliable, being based on solid and, on average, coherent evidence.

## 5. Conclusions

This systematic review selectively evaluated the impact of several lifestyle patterns on EAC risk. Despite the wealth of available literature on esophageal cancer and associated risk factors, no extensive overview focusing solely and specifically on EAC is available. This systematic review leads us to suggest that no single specific food is able to prevent disease (EAC), but rather a lifestyle pattern which takes into consideration other factors besides diet (as for instance socioeconomic status, smoking, alcohol, physical activity, and cooking processes).

Since prevention (both primary and secondary) remains the best option for esophageal adenocarcinoma, we need to provide patients and the high-risk population with comprehensible and easy to follow recommendations.

## Figures and Tables

**Table 1:** Inclusion and exclusion criteria for the studies’ review combining the PICOS framework.

Parameter	Inclusion	Exclusion
Population	Adult population, Male and female, Focusing on EAC alone.	Population with ESCC and EAC combined, esophageal and gastric cardia adenocarcinoma combined, only considered ESCC
Intervention	Administration of questionnaire evaluating, food frequency, dietary pattern, BMI, physical activity, smoking habit, alcohol consumption, sociodemographic characteristics	Medication or other intervention intended to reduce EAC risk
Control/ Comparison	Stratification according to dietary habits, dietary pattern, BMI, physical activity, smoking habit, alcohol consumption, sociodemographic characteristics	None
Outcomes	Risk of EAC	Risk of EAC and ESCC combined or ESCC alone Risk of EAC and gastric cardia adenocarcinoma combined
Study design	Epidemiologic studies (case-control, cross-sectional, or cohort studies), pooled analysis, meta-analysis.	Review article, expert opinion, commentary, article with no quantitative information or details, experimental animal models, genetic or immune-histochemical studies
Language filter	Only article in English language	Any other language
Time filter	From inception until May 2021	None

Abbreviations: EAC, esophageal adenocarcinoma; ESCC, esophageal squamous cells carcinoma; BMI, body mass index.

**Table 2:** All included studies (cohort, cross-sectional, and case-control), stratified by risk factors, direction of the association (beneficial, detrimental, or no effect), and quality evaluation. Numbers in square bracket refer to the number of references.

	High Quality (NOS ≥ 7) [References]			Medium/Low Quality (NOS < 7) [References]		
	Beneficial Effect	Detrimental Effect	No Effect	Beneficial Effect	Detrimental Effect	No Effect
Anthropometric measures						
Overweight/obesity (BMI ≥ 25)	-	[38,39,40,42,53,86,95,97,103,104,119,121,122,125,130,133]	[110,116,131]	-	[57,63,75,80,81,88,93,123]	-
Abdominal obesity						
High WC	-	[40,110,118,121,126,130,131]	-	-	[65]	-
High WHR	-	[121,126,130]	[32]	-	-	-
High hip-circumference	[131]	-	-	-	-	-
Physical activity	[89]	-	[110,111]	-	-	-
Dietary patterns						
“Healthy diet”	[117]	-	[33]	-	-	-
“Western diet”	-	[33,36,50]	-	-	-	-
“High carbohydrates”	-	-	-	[56,94]	-	-
“High fat”	-	-	[70,74,120]	-	[56]	-
“High proteins”	-	-	-	-	-	[56]
“High Dietary inflammation index”	-	[63,64]	-	-	-	-
“Plant-based”	[66,70,71]	-	-	-	-	-
“Animal-based”	-	[66,71]	-	-	-	-
Foods of plants origin and dietary fiber						
Vegetables	[84,87,129]	-	[106,108]	[80]	-	-
Fruits	[32,38]	-	[106,108,129]	-	-	-
Dietary fiber	[66,87,96]	-	-	[35,58]	-	-
Animal products						
Red meat	-	[72,112]	[96,101]	[92]	[90]	-
White meat	-	-	[101,112]	-	-	-
Processed meat	[109]	[112]	[96,101,109]	-	[90]	-
Total meat	-	-	[96,109]	-	-	-
Nonalcoholic beverages	-	-	-	-	-	-
Carbonated soft drink	-	[59]	[55,67,124]	-	-	-
Hot beverages	-	-	[124,135]	-	[48]	-
Vitamins, minerals, and other nutrients						
Polyphenols	[61,132]	-	[79] *	-	-	[62]
Vitamin C	[66,69,83,87]	-	-	[34]	-	-
Vitamin E	[51]	-	[69,83,99]	[34]	-	-
Vitamin A and Carotenoids	[66,83,87]	-	[69]	[35]	-	-
Folates	[66,82]	-	[134]	[35]	-	-
B Vitamins	[66]	-	[82,134]	-	-	-
Vitamin D and calcium	-	[68] ^+^	-	-	-	-
Heme iron	-	[73,112]	[115]		[92]	
Nonheme iron	[73,115]	-	-	-	-	-
Other compounds (Magnesium, Zinc, Selenium)	[128]	-	[41,69]	[35,94]	-	-
Dietary supplements	[51,69,87]	-	[99,102]	[54]	-	-
Cooking process and chemical modification during cooking						
Frying/broiling	-	-	-	-	-	[90]
Grilling/barbecuing	-	-	-	-	[90]	-
Acrylamide	-	-	[60]	-	-	-
Heterocyclic amines	-	[101]	-	-	-	[85]
Alcohol	-	-	[31,45,55,63,95,105,107,127]	-	[37,48]	[47,49,77,88]
Smoking	-	[32,45,46,63,95,103,105,110,127,136]	[107]	-	[37,44,47,48,76,78,80,88,93]	[49,54,77]
High socioeconomic factors	[46,52]	-	-	-	-	-

BMI: body mass index; NOS: Newcastle–Ottawa Scale; WC: waist circumference; WHR: waist/hip ratio. * Anthocyanidins reduced the risk; ‘only for current smokers; ^+^ only for vitamin D, but no association with calcium.

## Data Availability

All data are presented in the current manuscript.

## Supplementary Materials

The following are available online at www.mdpi.com/article/10.3390/nu13103525/s1, Table S1: Characteristics of included case-control studies, in alphabetical order, Table S2: Characteristics of included cohort and cross-sectional studies, in alphabetical order, Table S3: Quality assessment of case-control studies, using the Newcastle–Ottawa Scale (NOS), in alphabetical order. Table S4: Quality assessment of cohort studies, using the Newcastle–Ottawa Scale (NOS), in alphabetical order, Table S5: Preferred Reporting Items for Systematic Reviews and Meta-Analysis (PRISMA) checklist for abstract, Table S6: Preferred Reporting Items for Systematic Reviews and Meta-Analysis (PRISMA) checklist for reporting systematic reviews.
