# Supplementary material for: Lifestyle, WCRF/AICR Recommendations, and Esophageal Adenocarcinoma Risk: A Systematic Review of the Literature
Source: Nutrients. 2021 Oct 8;13(10):3525. doi: 10.3390/nu13103525 (PMC8538904; doi:10.3390/nu13103525)
Supplement: Supplementary file 1 [file nutrients-13-03525-s001.zip › Supplementary Tables_26092021.pdf]

Supplementary Table S1. Characteristics of included case-control studies, in alphabetical order.

Author Year [Ref]	Study Period / Country	Study Design	EAC Cases (n)	Control (n)	Aims	Data	Main Results
Anderson et al. 2009 [31]	2002-2004 / IE	Population-based case-control	227	260	To investigate relationship between alcohol intake and EAC.	Structured computerized interview	No association between total alcohol consumption and EAC.
Anderson et al. 2007 [32]	2002-2004 / IE	Population-based case-control	227	260	To investigate risk factors associated with EAC.	Structured interview	No relationship between WHP and EAC. Strong relationship between smoking and EAC.
Bahmanyar et al. 2006 [33]	1995-1997 / SE	Case-control	185	815	To examine the association of dietary patterns and the development of EAC.	Structured FFQ, including 63 food and beverage items of interest evaluating 20 y before interview.	The healthy diet pattern was associated with moderately non-statistically significant risk reduction.
Bollschweiler et al. 2002 [34]	1997-2000 / DE	Pilot-study	47	50	To analyze the influence of daily vitamin consumption on the frequency of EAC.	Structured questionnaire	EAC risk reduction with an intake of 13mg/day of vitamin E and >100mg/day of vitamin C.
Chen et al. 2002 [35]	1992-1994 / US	Population-based case-control	124	449	To investigate the relationship between nutrient intake and EAC.	Telephone interview. Short Health Habits and History Questionnaire.	Significant inverse association with EAC risk and vitamin A, β-cryptoxanthin, riboflavin, folate, zinc, dietary fiber, protein, and carbohydrate. Positive association between saturated fat intake and risk of EAC.
Chen et al. 2002 [36]	1992-1994 / US	Population-based case-control	124	449	To evaluate potential roles of dietary patterns in the etiology of EAC.	Telephone interviews. Short Health Habits and History Questionnaire.	Consumption of dairy products, fish, all vegetables, citrus fruits and juice, and dark bread reduced EAC risk. Positive association with gravy intake.

Chen et al. 2011 [37]	2002-2006<br>CN	Case-control	98	294	Relation between smoking and alcohol consumption and EAC risk.	Interview survey	Heavy alcohol consumption and heavy smokers increase risk of EAC independently from the duration.
Cheng et al. 2000 [38]	1993-1996<br>UK	Population-based case-control	74	74	Relation between fruit and vegetables intake, BMI and breast feeding and EAC risk.	Dietary questionnaire for recent diet (3 years before) and for past diet (30 year before).	Statistically significant inverse relation between fruit intake and EAC risk. Statistically significant positive association between higher BMI and risk of EAC.
Chow et al. 1998 [39]	1993-1995<br>US	Case-control	292	695	To evaluate the possible role of excess weight as a risk factor for EAC.	Structured interview to elicit information up to 1 year prior to diagnosis for case patients and date of interview for control subjects.	BMI showed excess risks, significantly modified by age. Largest BMI-related increase in risk was found among non-smokers, followed by current smokers and then former smokers.
Corley et al. 2008 [40]	2006<br>US	Nested case-control	101	2,800	To assess the association between abdominal obesity, BMI, and the risk of EAC.	Instructed examiner collected anthropometric data. Questionnaire regarding potential confounders such as GERD symptoms within the last 6 months, food intake, drug use hiatal hernia diagnosis, smoking status, recent alcohol use, race.	Abdominal diameter is an independent risk factor, even after adjustment for BMI, suggesting that the association between abdominal diameter and cancer risk was not solely mediated by a higher BMI.
Dai et al. 2016 [41]	2002-2005<br>IE	Population-based case-control	218	252	To evaluate the association between Mg intake and EAC risk.	Trained interviewers using an electronic questionnaire investigating lifestyle, medication, and co-morbidities. Dietary	No significant association between intake of Mg and risk for EAC or a significant interaction between intakes of vitamin D and Mg.

						intake was assessed using a 101-item FFQ, adapted from a version of the EPIC-FFQ.	
de Jonge et al. 2006 [42]	2003-2005<br>NL	Hospital-based case-control	91	244	To identify risk factors to differentiate between Barrett's Esophagus patients with low or high risk of EAC.	Non-validated questionnaire.	Tobacco smoking and BMI >25 Kg/m2 increased the risk of EAC.
Drahos et al. 2016 [43]	1992-2012<br>UK	Population-based case-control	592	2,901	To investigate MetS in relation to EAC.	GPRD	No association between MetS and risk of EAC.
Duan et al. 2009 [44]	1992-1997<br>US	Population-based case-control	220	1,356	Relation between passive smoking and risk of EAC.	In-person interview with structured questionnaire.	Current smokers were at increased EAC risk. Exposure to at least one smoker during adulthood was associated with an elevated risk of EAC.
Engel et al. 2003 [45]	1993-1995<br>US	Population-based case-control	293	695	To identify population attributable risk (PAR) for EAC.	Trained interviewers administered structured, in-person interviews; FFQ; Blood samples.	Increasing levels of education or income decreased the risk of EAC. Smoking increased the risk. Beer and hard liquor were not associated. Wine decreased the risk of EAC.
Gammon et al. 1997 [46]	1993-1995<br>US	Multicenter case-control	293	695	To identify EAC risk factors.	Face-to-face interviews with structured questionnaire.	Doubled risk of EAC among current and former smokers. No association between beer and hard liquor intake. 40% risk reduction was associated with wine drinking.
Gao et al. 1994 [47]	1990-1993<br>CN	Case-control	51	1,552	Relation between alcohol consumption, smoking habit and risk of EAC.	Structured standardized questionnaire.	Smoking more than 30 cig/day was associated with an increased risk of EAC. Alcohol did not increase EAC risk.
	1989-1991	Case-control	56	200			

Garidou et al. 1996 [48]	GR				Relation between lifestyle and EAC risk.	Structured standardized questionnaire; FFQ.	Higher education was associated with a decreased risk of EAC. Smoking and alcohol were risk factors for EAC. Hot or very hot beverages were associated with a higher risk of EAC.
Hashibe et al. 2007 [49]	2000-2002<br>RO, RU, CZ, PL	Multicenter case-control	35	1,114	Relation between cigarette smoking, alcohol intake and EAC risk.	Face-to-face interview with structured questionnaire.	No statistical association between cigarette smoking, alcohol consumption and EAC.
Ibiebele et al. 2012 [50]	2002-2005<br>AU	Population-based case–control	299	1,507	To investigate the role of dietary patterns in EAC risk.	Self-administered questionnaire (general data); 139-item semi-quantitative FFQ.	Meat-and-fat dietary pattern had a 2-fold increased risk of EAC. High-fat dairy foods play a dominant role in the association between the meat-and-fat pattern and risk of EAC.
Ibiebele et al. 2013 [51]	2002-2005<br>AU	Population-based case–control	299	1,507	Relation between dietary antioxidant intake and risk of EAC.	135-item semi-quantitative FFQ.	Inverse association between vitamin E intake, high antioxidant and EAC.
Jansson et al. 2005 [52]	1995-1997<br>SE	Population-based case-control	189	820	To examine the association between socioeconomic status and development of EAC.	Questionnaire to collect data on occupational history, educational level and other socioeconomic dimensions.	Low socioeconomic status increased EAC risk. However, the association is not statistically significant in the adjusted model.
Lagergren et al. 1999 [53]	1995-1997<br>SE	Population-based case-control	189	820	To examine the association between BMI and EAC.	Face-to-face interview.	The association between BMI and EAC is highly significant.
Lagergren et al. 2000 [54]	1995-1997<br>SE	Population-based case-control	189	820	To examine the association between smoking, snuff, and alcohol use and development of EAC.	Face-to-face interview.	Weak or absent association considering the interaction between tobacco smoking and alcohol and EAC risk.
	1995-1997		189	820			

Lagergren et al. 2006 [55]	SE	Population-based case-control			Association between carbonated drink intake and EAC.	Validated FFQ that surveyed intake of beverages and dietary patterns 20 years earlier.	No association between carbonated soft drinks or low-alcohol beer and EAC.
Lagergren et al. 2013 [56]	1995-1997<br>SE	Population-based case-control	189	820	Association between the proportions of carbohydrates, fat, and protein and the risk of EAC.	FFQ adapted from a validated standard questionnaire.	A high dietary proportion of carbohydrates decreased the risk of EAC, a high portion of fat increased the risk, while a high proportion of protein did not influence the risk of EAC.
Lahmann et al. 2014 [57]	1995-1997<br>SE	Population-based case-control	189	816	Relation between BMI and EAC.	Interview	BMI is a strong and independent risk factor for EAC.
Lahmann et al. 2014 [58]	2002-2005<br>AU	Population-based case-control	299	1,507	Association between GI, GL, total carbohydrate intake and risk of EAC.	135-item semi-quantitative FFQ.	GI, GL and total carbohydrate intake did not increase the risk of EAC. Strong inverse association between EAC risk and fiber intake.
Li, N. et al. 2017 [59]	1993-1195<br>US	Population-based case-control	500	2,027	Association between the intake of sweetened beverages and the risk of EAC.	104-item FFQ.	The intake of sweetened beverages is associated with an increased risk of EAC.
Lin et al. 2011 [60]	1994-1997<br>SE	Population-based case–control	189	820	Association between dietary acrylamide intake and risk of EAC.	FFQ concerning the habitual intake of 63 foods and beverages as recalled from 20 years before the interview.	EAC risk moderately increased among persons with higher exposure to dietary acrylamide, without dose-response effects.
Lin et al. 2014 [61]	1995-1997<br>SE	Population-based case–control	181	806	To test whether dietary lignans, quercetin, and resveratrol synergistically decrease the risk of EAC.	FFQ concerning the habitual intake of 63 foods and beverages as recalled from 20 years before the interview.	Dietary pattern characterized by the intake of lignans, quercetin and resveratrol have a protective role in the EAC onset.

Lin et al. 2012 [62]	1994-1997<br>SE	Population-based case–control	181	806	Association between dietary intake of lignans and risk of EAC.	Personal interview; FFQ with 63 foods and beverages, assessing dietary habits 20 y before the interview.	High dietary intake of lignans reduced the risk of AEC.
Lindblad et al. 2005 [63]	1994-2001<br>UK	Prospective nested case-control	287	10	To prospectively assess the influence of BMI, tobacco, and alcohol on the occurrence of EAC.	Information recorded at least two years before the date when the tumor was first recorded or when the review revealed an earlier date of diagnosis.	BMI is a risk factor for EAC, in a dose-response relation. Current smokers but not former smokers were at an increased risk of EAC. Alcohol was not associated with risk of EAC.
Lu et al. 2016 [64]	1994-1997<br>SE	Population-based case–control	181	806	To assess the association between diet-related inflammation and EAC risk.	Face-to-face interview; FFQ with 63 foods and beverages, assessing dietary habits 20 y before the interview.	Significant positive associations between DII and EAC.
Massl et al. 2014 [65]	2003-2011<br>NL	Case-control	175	251	Relation between visceral adipose tissue measured by CT and EAC risk.	CT scan	Visceral adipose tissue is a risk factor in the development of EAC. Visceral adipose factor seems to be more important than obesity per se.
Mayne et al. 2001 [66]	1993-1995<br>US	Multicenter case-control	282	687	Associations between nutrient intake and risk of EAC.	Face-to-face interviews; 104-item FFQ.	Nutrients from plant foods (fiber, β-carotene, folate, vitamin C, and B6) were associated with a reduced risk of EAC. Nutrients from animal foods (dietary cholesterol, animal protein, and vitamin B12) were associated with an increased risk of EAC
Mayne et al. 2006 [67]	1993-1995<br>US	Multicenter, population-based case - control	282	687	Association between CSD consumption and EAC.	In-person structured questionnaire asking about the frequency of “diet soft drinks or soda” and “regular soft drinks or soda”, not diet consumption,	High CSD consumption was associated with a reduced risk of EAC in men and women. High consumption of “diet” CSDs compared to “regular” CSDs was associated with a statistically significant lower risk of EAC.

						3 – 5 years before diagnosis (case) or interview (control).	
Mulholland et al. 2011 [68]	2002-2005<br>IE	Population based case-control	218	252	Association between vitamin D, Ca, and dairy product intake and the risk of EAC.	101-item EPIC-FFQ adapted for the Irish population; self-reported BMI.	A high dietary vitamin D intake is associated with higher EAC risk. Ca intake was not associated with EAC.
Murphy et al. 2010 [69]	2002- 2004<br>IE	Case-control	224	256	Association between vitamin C, E, carotenoids, selenium, copper, and zinc and risk of EAC.	Interview; EPIC-FFQ adapted for the Irish population.	The overall antioxidant index is associated with a lower risk of EAC. Vitamin C intake reduces EAC risk in smokers. No association between carotenoids, vitamin E, zinc, and cooper and risk of EAC.
Navarro Silvera et al. 2014 [72]	1993- 1995<br>US	Multicenter population-based case-control	282	687	Relation between diet, lifestyle and EAC risk.	Structured in-person questionnaire; 104-item FFQ evaluating diet 3-5 years before EAC diagnosis or before the interview for controls.	Red meat was associated with an increased risk of EAC. Non-citrus fruits reduced risk of EAC.
Navarro Silvera et al. 2008 [70]	1993- 1995<br>US	Multicenter population-based case-control	282	687	Association between different food groups and risk of EAC.	Structured in-person questionnaire; 104-item FFQ evaluating diet 3-5 years before EAC diagnosis or before the interview for controls.	Fruits, yellow and dark green vegetables, and whole grain are inversely associated with EAC risk. Red meat, dairy, and high-fat foods was associated with an increased risk of EAC.
Navarro Silvera et al. 2011 [71]	1993- 1995<br>US	Multicenter population-based case-control	282	687	Relation between dietary patterns, lifestyle and EAC risk.	104-item structured in-person FFQ evaluating diet 3-5 years before EAC diagnosis or before the interview for controls.	Significant inverse association between the pattern of fruits/vegetables and EAC risk. Positive significant association between meat/nitrites and EAC.
O'Doherty et al. 2010 [73]	2002-2005<br>IE	Case-control	224	256	Relation between Iron intake and risk of EAC.	Structured computerized 101-item EPIC-FFQ,	Total iron and non-heme iron and vitamin C were inversely associated

						(adapted for the Irish population) relating to a period of 5 years before interview.	with EAC. Heme iron was positively associated with EAC
O'Doherty et al. 2011 [74]	2002-2005<br>IE	Case-control	224	256	Association between dietary fat and meat intake with EAC.	Structured computerized 101-item EPIC-FFQ, (adapted for the Irish population) relating to a period of 5 years before interview.	High intakes of total fat, saturated fat, monounsaturated fat and fresh red meat were associated with higher risk of EAC.
Olsen et al. 2011 [75]	2002-2005<br>AU	Population-based case-control	364	1,580	Population attributable fractions of EAC associated with BMI and smoking.	Self-administered questionnaire	BMI≥30 and frequent acid reflux accounted for the greatest proportions of EAC. Total Population Attributable Fraction (PAF) associated with smoking, BMI, and symptoms of GERD was higher for men (78% v. 59% women), although the difference was not significant.
Pandeya N et al. 2008 [76]	2002-2005<br>AU	Population-based case-control	367	1,580	Association of duration, intensity, and smoking quantity with EAC.	A self-completed health and lifestyle questionnaire.	Smoking and duration of smoking were significantly associated with EAC; however, intensity was not. Time since quitting was independently associated with an approximate 15% risk reduction per decade.
Pandeya et al. 2009 [77]	2002-2005<br>AU	Population-based case-control	365	1,580	Relation between alcohol intake and EAC risk.	A self-completed health and lifestyle questionnaire.	No association between alcohol and EAC. No significant interaction between smoking and alcohol. Beer was not associated with EAC risk. Modest intake of wine reduced EAC risk.
Pandeya et al. 2010 [78]	2002-2005<br>AU	Population-based case-control	365	1,580	Relation between smoking and EAC risk in patients with GERD.	A self-completed health and lifestyle questionnaire.	GERD was associated with a 6.4-fold increase in EAC risk. Heavy smokers had a markedly high EAC risk.
	1993-1995		274	662			

Petrick et al. 2015 [79]	US	Multicenter population-based			Association between intakes of total flavonoids and lignans, and the incidence of EAC.	104-item face-to-face structured FFQ evaluating diet 3-5 years before EAC diagnosis or before the control interview.	Little or no consistent association was found between total flavonoid intake and the incidence of EAC. Anthocyanidins was associated with a reduction in the risk of EAC and mortality from.
Pohl et al. 2013 [80]	2005-2009<br>DE	Case-control	100	No-GERD (n=113) GERD (n=188)	To examine at what stage known risk factors exert their influence toward EAC progression.	Standardized questionnaire	Increasing BMI at age 40 showed a small, but significant, association with EAC. Fruits and vegetables showed a strong protective effect. Duration of smoking and timing of the largest meal during the day was not associated with EAC. Any history of smoking was associated with a risk of EAC.
Ryan et al. 2006 [81]	1994-2004<br>IE	Prospective case-control	936	893	Relation between BMI and obesity and EAC risk.	Registered dietitian assessed every patient individually.	A high pre-illness BMI significantly raises the risk of EAC, with a dose-dependent relationship.
Sharp et al. 2013 [82]	2002-2005<br>IE	Case-control matched study	223	223	Association between dietary folate and risk of EAC.	Structured interviews and completed food-frequency questionnaires.	EAC risk decreased with increasing folate and Vitamin B-6 intake. Vitamin B-12 intake increased EAC risk.
Terry et al. 2000 [83]	1995-1997<br>SE	Nationwide, population-based case-control	185	815	Relation between vitamin C, β-carotene, and α-tocopherol intake and risk of EAC.	63-item FFQ in computer-aided face-to-face interviews. Usual intake 20y before the interview.	Vitamin C and β-carotene intake was inversely associated with EAC risk. α-tocopherol was not associated. Parallel dietary intake of vitamin C, Alpha-tocopherol, and β-carotene decrease the risk of EAC. No risk reduction with vitamin supplementation.
Terry et al. 2001 [84]	1995-1997<br>SE	Nationwide, population-based case-control	185	815	Association between fruit and vegetable consumption and the risk of EAC.	63-item FFQ in computer-aided face-to-face interviews. Usual intake 20y before the interview.	Fruits and vegetables reduced the risk of EAC. There were no significant associations between any specific fruit or vegetable and EAC.
Terry et al. 2003 [85]	1995-1997<br>SE	Nationwide, population-	185	815	Relation between heterocyclic amine	63-item FFQ in computer-aided face-to-face interviews;	No association was found between heterocyclic amine intakes and the risk of EAC.

		based case-control			intake and risk of EAC	usual intake 20y before the interview; questions on specific foods and cooking methods.	
Thrift et al. 2014 [86]	BEAGESS (data from 14 epidemiological studies conducted in Western Europe, Australia, and North America)	Case-control Mendelian approach	999	2,169	To improve the precision of causal estimates between BMI and EAC.	Genetic data sourced from genome-wide association studies (GWAS).	EAC risk increased by 16% per BMI increase of 1 kg/m2.
Tzonou et al. 1996 [87]	1989-1991<br>GR	Case-control	56	200	To identify dietary risk factors for EAC.	115-item validated semi-quantitative FFQ.	Added polyunsaturated fats are positively associated with EAC risk. Intake of vegetables, fruits, vitamin C, crude fiber, and vitamin A was inversely associated with EAC.
Veugelers et al. 2006 [88]	2001-2003<br>CA	Prospective hospital–based case-control	57	102	Relation between obesity, diet, smoking, and alcohol consumption and EAC risk.	102-point face- to- face interview; 150-item structured FFQ.	BMI and smoking were risk factors for EAC. Vitamin C was associated with a lower risk of EAC. Liquor was not a risk factor for EAC.
Vigen, C. et al. 2006 [89]	n.a.	Population-based case-control	212	1,330	Relation between occupational physical activity and EAC.	In-person interviews and total lifetime occupational activity were calculated using US Census job codes classified as sedentary, or moderately or highly physically active.	EAC risk decreases with an increase in the Total Activity Index (OR = 0.67, 95% CI = 0.38,1.19 for highest versus lowest quartile).
Ward et al. 1997 [90]	1992-1994<br>US	Population-based case-control	137 whites only	502	Association between meat and gravy intake, meat	Telephone interview; HHHQ	Processed and red meats increased risk of EAC. Consumption of gravy ≥4 times/week was associated with more

					cooking methods and doneness preference, and the risk of EAC.		than a 2-fold increased risk of EAC. Frying or broiling were not associated with risk of EAC. Barbecuing were associated with EAC.
Ward et al. 2012 [92]	1992-1994<br>US	Population-based case-control	124 whites only	449	Relation between heme and total iron intake from meat, and the risk of EAC using a new database of heme iron levels developed at the NCI.	Short HHHQ with addition of foods high in nitrate/nitrite and questions about meat cooking methods and doneness preferences for beef, pork, and chicken.	High intake of red meat was associated with EAC. The risk increased with increasing quartiles of heme and total iron from meat, with a stronger association for heme iron.
Ward et al. 2008 [91]	1992-1994<br>US	Population-based case-control	137	503	To assess the relationship between drinking water, nitrate and nitrite intake, and the risk of EAC.	Questions about residential and water source history, demographic factors, tobacco and alcohol use, pesticide use by farmers, occupational history medical conditions, and medication use. Short HHHQ with addition of foods high in nitrate/nitrite.	No association between intake of nitrate from public water and risk of EAC. Dietary nitrate and nitrite from animal food were significantly associated with EAC risk.
Whiteman et al. 2008 [93]	2001-2005<br>AU	Population-based case–control	367	1,580	To measure the relative risks of EAC associated with measures of obesity and their interactions with age, sex, GERD symptoms, and smoking.	Self-completed, mailed questionnaires; telephone interview.	BMI increased the risk of EAC, particularly in men and among those aged <50 years. Smoking is associated with higher risk of EAC with increasing BMI.
Wolfgarten et al. 2001 [94]	1997-1998<br>DE	Case-control	40	100	Relation between nutrient intake,	2-page dietary questionnaire.	Sodium, potassium, phosphate, zinc, and selenium were not associated

					dietary patterns and EAC risk.	Personal interview on nutritional habits using the EBIS computer program.	with EAC. > 1,300 mg/day of calcium, > 500 mg/day of magnesium, and 18 mg/day of iron were inversely correlated with EAC. Dietary fiber, carbohydrates, and fruits were significantly higher in controls than in patients with EAC.
Wu et al 2007 [96]	1992-1997<br>US	Case-control	206	1,308	Relation between dietary fat and fiber intake and risk of EAC. To investigate risk associated with the intake of total meat, red meat, poultry, fish/shellfish, and processed meat and EAC.	124-item food and beverage in-person interviews. Question on the use of dietary supplements also added.	Total fat, saturated and monounsaturated fat, increased the risk of EAC, but not the polyunsaturated fat. Total fiber reduced the risk. Total meat or processed meat intake, poultry and fish/shellfish were unrelated to EAC risk.
Wu et al. 2001 [95]	1992-1997<br>US	Large population-based case-control	222	1,356	Relation between BMI, alcohol, and cigarette smoking and EAC risk.	Structured questionnaire	Smoking was a risk factor for EAC. Smoking cessation did not reduced the risk. Alcohol was not associated with EAC risk. BMI at ages 20 and 40 years increased the risk in a dose-dependent manner.
Abbreviations: AU, Australia; BEAGESS, Barrett's and Esophageal Adenocarcinoma Genetic Susceptibility Study; BMI, Body Mass Index; CA, Canada; CI, Confidence Interval; CN, China; CSDs, Carbonated Soft Drinks; CT, Computed Tomography; CZ, Czech Republic; DE, Germany; DII, Dietary Inflammation Index; EAC, Esophageal Adenocarcinoma; EPIC, European Prospective Investigation into Cancer and Nutrition; FFQ, Food Frequency Questionnaire; GERD, Gastroesophageal Reflux Disease; GI, Glycemic Index; GL, Glycemic Load; GPRD, General Practice Research Database; GR, Greece; HHHQ, Health Habits and History Questionnaire; IE, Ireland; LBS, Pounds; MetS, Metabolic Syndrome; Mg, Magnesium; NCL, National Cancer Institute; NL, Netherlands, OR, Odds Ratio; PL, Poland; RO, Romania; RU, Russia; RR, Relative Risk; SE, Sweden; UK, United Kingdom; US, United States, y, years.							

Supplementary Table S2. Characteristics of included cohort and cross-sectional studies, in alphabetical order.

Author Year [Ref]	Study Period / Country	Study Design	EAC Cases (n)	Study Population (n)	Aims	Data	Main Results
Abnet et al. 2008 [97]	1995-1996 (7y FU) / US	Cohort (NIH-AARP Diet and Health Study)	371	480,475	Evaluating the association between BMI and risk of developing EAC.	Mailed questionnaire on demographics, dietary intake and health-related behaviors.	BMI categories greater than normal had significant and progressively greater risk.
Allen et al. 2009 [98]	1996-2001 (7y FU) / UK	Cohort (The Million Women Study)	226	1,280,296	Association between moderate alcohol intake and risk of EAC in women.	Questionnaire without any other details.	Alcohol was not associated with EAC.
Carman et al. 2009 [99]	1995-1996 (7y FU) / US	Cohort (NIH-AARP Diet and Health Study)	382	492,559	Association of dietary α-tocopherol, γ-tocopherol, and supplemental vitamin E with the risk of EAC.	124-item FFQ	Dietary α-tocopherol and γ-tocopherol were not associated with EAC. Supplemental Vitamin E was no associated with EAC.
Cook et al. 2013 [100]	1995-1996 (10y FU) / US	Cohort (NIH-AARP Diet and Health Study)	631	303,033	To investigate the relationship between physical activity, sedentary behavior, and EAC.	Self-administered baseline questionnaire. Mailed risk factor questionnaire (after 6 months from baseline).	Inverse association between sedentary behavior and EAC.
Cross et al. 2011 [101]	1995-1996 (10y FU) / US	Cohort (NIH-AARP Diet and Health Study)	630	494,979	To investigate the relationship between meat and meat-related variables and EAC.	Self-administered baseline questionnaire. 124-item FFQ. Mailed risk factor questionnaire (after 6 months from baseline).	Red meat, white meat and processed meat consumption, was not associated with EAC. A high HCA intake was associated with borderline statistically increased risk of EAC. Positive association between heme iron intake and EAC. No association

							between B[a]P, nitrate, or nitrite and EAC.
Dawsey et al. 2014 [102]	1995-1996 (10y FU) US	Cohort (NIH-AARP Diet and Health Study)	625	490,593	To investigate the association between multivitamins and other supplements and EAC.	Self-administered baseline questionnaire.	Multivitamin and calcium supplements increased EAC risk in smokers. Iron supplement reduced the EAC risk.
de Jonge et al. 2007 [103]	2002-2005 NL	Cross-sectional	126	226	To investigate the relation between environmental risk factors and EAC.	Standard questionnaire	BMI and a smoking habit increased EAC risk in males.
Engeland et al. 2004 [104]	1963-2001 NO	Cohort	575	2,001,617	To investigate the association between BMI, stature, and cancer.	Height and weight measured by trained staff.	Being overweight and obese increased EAC risk.
Freedman et al. 2007 [105]	1995-1996 (5y FU) US	Prospective cohort (NIH–AARP Diet and Health Study)	205	474,606	To investigate the association between tobacco, alcohol, and the risk of EAC.	Self-administered validated questionnaires concerning alcohol intake and tobacco.	Current and former cigarette smoking increased risk of EAC. Alcohol was not associated with EAC.
Freedman et al. 2007 [106]	1995-1996 (5y FU) US	Cohort (NIH-AARP Diet and Health Study)	213	490,802	To investigate the association between fruit and vegetable consumption and the risk of EAC.	Self-administered baseline questionnaire 124-item FFQ	Fruit and vegetable were not associated with EAC. Spinach reduced EAC risk.
Gatenby et al. 2008 [107]	1996-2004 UK	Hospital-based prospective cohort		1,651	To investigate the association between demographic characteristics, lifestyle, and EAC risk.	Information from patient hospital records.	No association between gender, smoking habits, alcohol consumption and the incidence of EAC.
Gonzalez et al. 2006 [108]	1992-1998 DK, FR, DE, GR, IT, NL,	EPIC - Cohort	65	481,518	To describe the effect of fruit and vegetable	12-month previous country-	Negative with a non-significant association for vegetable intake and citrus intake.

	NO, ES, SE, UK				intake on the risk of EAC.	specific validated FFQ.	
Gonzalez et al. 2006 [109]	1992 -2002<br>DK, FR, DE, GR, IT, NL, ES, SE, UK	EPIC - Cohort	65	481,518	To investigate the risk of EAC associated with the consumption of meat and processed meat.	12-month previous country-specific validated FFQ.	Positive but no statistically significant association between EAC risk and total and processed meat intake. Poultry reduced the risk of EAC.
Hardikar et al. 2013 [110]	1995-2009<br>US	Prospective cohort (Seattle Barrett's Esophagus Study – SBES)	45	411	Association between smoking, school, and obesity and EAC.	Structured personal 45-minute interview.	Smoking duration increased EAC risk. Alcohol and BMI did not.
Huerta et al. 2010 [111]	1991-2005<br>DK, FR, DE, GR, IT, NL, ES, SE, UK	Prospective cohort (EPIC)	80	420,449	To investigate the association between physical activity and EAC.	Validated questionnaires; CPAI.	Physical activity was not associated with EAC.
Jakszyn et al. 2013 [112]	FR, IT, ES, UK, NL, SE, DE, DK	Prospective cohort (EPIC)	137	481,419	To investigate the association between the intake of different types of meats, heme iron intake and EAC risk.	Validated center-specific questionnaires.	Heme iron and processed red meat increased EAC risk, but not white and unprocessed meat (based on tertiles of intake), but not when continuous variable was considered.
Ji et al. 2017 [113]	1973-2010<br>SE	Retrospective cohort	145	420,489	To investigate the association of alcohol consumption with EAC.	Swedish Hospital Discharge Register and Outpatient Register; Crime Register; Prescription Drug Register.	Subjects with alcohol use disorders (heavy alcohol drinkers) had an increased risk of EAC.
Keszei et al. 2012 [114]	1986-2002<br>NL	Case-cohort<br>The Netherlands Cohort Study (NLCS)	145	3,921 (sub-cohort)	To investigate the association between red and processed meat and the risk of EAC.	Self-administered baseline questionnaire with a 150-item questionnaire on	No association between EAC risk and red meat and processed meat intake.

						food and beverage consumption during the year prior to the start of the study.	
Keszei et al. 2013 [115]	1986-2002<br>NL	Case-cohort The Netherlands Cohort Study (NLCS)	151	4,032 (sub-cohort)	Relation between the risk of EAC and dietary intake of N-nitroso-dimethyl-amine, heme iron, nitrite, and nitrate.	Self-administered baseline questionnaire with a 150-item questionnaire on food and beverage consumption during the year prior to the start of the study.	No associations between N-nitroso compounds and EAC risk.
Levi et al. 2013 [116]	1967-2006<br>IL	Cohort	28	1,088,242	To investigate the association between BMI in late adolescence, SES and ethnic factors and EAC incidence.	Measured height and weight; Israeli Central Bureau of Statistics; Israel National Cancer Registry.	Risk of EAC was not significantly increased in subject with BMI ≥85th percentile. No association between SES, ethnicity, and EAC.
Li et al. 2013 [117]	1995-2006<br>US	Cohort (NIH-AARP Diet and Health Study	633	494,968	To investigate the association between HEI-2005, aMED and the risk of EAC	124-food item FFQ; HEI-2005 aMED; Social Security Administration; Death Master File; Cancer registers.	Higher HEI-2005 was significantly associated with a reduced risk of EAC. No association between the aMED score and the risk of EAC.
Lin et al. 2015 [118]	1995-1997<br>2006-2008<br>NO	Cohort CONOR and HUNT3	62	192,903	To investigate the role of the metabolic syndrome and WC.	Anthropometric data measured objectively.	MetS was not associated with EAC, whilst a high WC was associated with an increased risk of EAC.
	1986-1999	Prospective cohort	133	4,552 sub-cohort		Self-administered questionnaire on	BMI was associated with EAC risk. Change in BMI during

Merry et al. 2007 [119]	NL	(The Netherlands Cohort Study on Diet and Cancer)			To investigate the association between BMI and EAC risk.	usual dietary intake, anthropometry, smoking habits, physical activity, education, and history of cancer.	adulthood was positively associated with the risk of EAC. No association with BMI in early adulthood.
O'Doherty et al. 2012 [120]	1995-2006<br>US	Cohort (NIH-AARP Diet and Health Study	630	494,978	To investigate the association between total fat and fat subtype intake and EAC.	Baseline questionnaire; 124-food item FFQ.	No consistent associations between total fat intake and fat subtypes with risk of EAC. Protective role of polyunsaturated fat intake in subjects with a normal BMI.
O'Doherty et al. 2012 [121]	1995-2006<br>US	Cohort (NIH-AARP Diet and Health Study	253	218,854	To investigate the relation between height, BMI, and abdominal obesity with EAC.	Baseline questionnaire; Risk factor questionnaire.	BMI≥35, WC and WHR increased the risk of EAC.
Petrick et al. 2017 [122]	1995-1996<br>US	NIH-AARP Diet and Health Study and Prostate, Lung, Colorectal, and Ovarian (PLCO) Cancer Screening Trial	633	409,796	To assess the effects of adiposity over the life course and EAC.	Self-reported anthropometric data.	Being overweight in early adulthood and weight gain later in life were also associated with an increased risk of EAC.
Reeves et al. 2007 [123]	1996-2001 (5.4y FU)<br>UK	Million Women Study	150	1,222,630	To examine the relation between BMI and EAC.	Self-reported anthropometric data.	Increasing BMI is associated with a higher risk of EAC.
Ren et al. 2010 [124]	1995-1996 (13y FU)<br>US	NIH-AARP Diet and Health Study	305	566,407	To investigate the relation between hot beverages, tea, coffee, carbonated soft drinks, and EAC.	Baseline questionnaire; Risk factor questionnaire.	No significant association with hot beverages, tea, coffee, and carbonated soft drinks.
	1971-1999	Cohort	82	362,552 males			Hihg BMI increased risk of EAC.

Samanic et al. 2006 [125]	SE				To examine the relationship between being overweight, obesity, and EAC risk.	Measured weight and height. Population-based Swedish cancer registry.	
Sanikini et al. 2020 [126]	10 European countries (DK, FR, DE, GR, IT, NO, ES, SE, NL, UK)	Cohort EPIC	220	476,16	To investigate anthropometric factors in relation to EAC.	Anthropometric measurements were taken at recruitment by trained health professionals.	WC and WHR were associated with a higher risk of EAC in both men and women.
Steevens et al. 2010 [127]	1986-2002<br>NL	Cohort (Netherlands Cohort Study on diet and cancer)	145	3,962 sub-cohort	To investigate the association between alcohol consumption, cigarette smoking, and the risk of EAC.	Self-administered questionnaire with a 150-item food FFQ.	No association between total alcohol and type of alcoholic beverage consumption and EAC risk. Frequency and pack-years of smoking were independently associated with risk of EAC. Risk reduction for smoking cessation and EAC was statistically significant.
Steevens et al. 2010 [128]	1986-2002<br>NL	Cohort (Netherlands Cohort Study on diet and cancer)	129	2,072 sub-cohort	To prospectively investigate the association between prediagnostic toenail selenium levels and risk of EAC.	Self-administered questionnaire with a 150-item FFQ and questions on cancer risk factors (smoking habits, alcohol consumption, height, and weight). Toenail clippings for selenium determination by	Inverse association between selenium status and risk of EAC in subgroups (women; never smokers; low antioxidant consumers).

						neutron activation analysis of the 77mSe isotope.	
Steevens et al. 2011 [129]	1986-2002<br>NL	Cohort (Netherlands Cohort Study on diet and cancer)	144	4,035 sub-cohort	To prospectively investigate the role of vegetable and fruit consumption in the development of EAC.	National Cancer Registry Nationwide Network; Registry of Histopathology and Cytopathology in the Netherlands; Self-administered questionnaire with a 150-item food FFQ.	Significant inverse associations were observed for raw vegetables, citrus fruits and EAC risk. Total fruit consumption was associated with a non-significant decrease in EAC risk. In current smokers only, vegetables were inversely associated with EAC risk.
Steffen et al. 2009 [130]	1992-2007<br>DK, DE, IT, NL, ES, SE, UK	EPIC - Cohort	124	391,456 Sub-cohort	To evaluate the relation between body height and general and abdominal obesity, with the incidence of EAC.	Directly measured weight, height, waist circumference, and hip circumference; non-dietary questionnaires for lifestyle and health-related information; country-specific FFQ.	BMI, high WC, and high WHR were statistically positively related to the risk of EAC. Body height was not associated with the risk of EAC. BMI and WC may be strongly associated with the risk of EAC in smokers, rather than in non-smokers.
Steffen et al. 2015 [131]	1993-2008<br>DK, DE, IT, NL, ES, SE, UK	EPIC - Cohort	88	346,544 sub-cohort	Association of anthropometric measures with risk of EAC.	Directly measured weight, height, waist circumference, and hip circumference; Lifestyle	BMI was unrelated to EAC. WC showed a strong positive association with EAC risk. Hip circumference (HC) was inversely related to EAC after controlling for WC Protective effect of gluteofemoral

						questionnaires for lifestyle and health-related information; country-specific FFQ.	(subcutaneous) adipose tissue in EAC.
Vermeulen et al. 2013 [132]	1992-2010<br>DK, FR, DE, GR, IT, NO, NL, ES, SE, UK	EPIC - Cohort	142	477,312	To investigate the association between dietary flavonoid intake and EAC risk.	Validated dietary country-specific questionnaires.	No statistically significant association between any flavonoid subclass and EAC.
Xiao et al. 2014 [134]	1995-2006<br>US	Cohort (NIH-AARP Diet and Health Study)	574	492,292	To investigate the association between folate, methionine, vitamin B6, and vitamin B12 intake and EAC risk.	Self-administered 124-item FFQ.	Higher intakes of folate, methionine, vitamin B6, or vitamin B12 were not associated with EAC risk. No association between total folate (diet+supplements) intake and EAC risk.
Yates et al. 2014 [133]	1993-2008<br>UK	EPIC-Norfolk Cohort	66	24,068	To investigate the relation between smoking, BMI, alcohol consumption and EAC risk.	EPIC questionnaire.	BMI greater than 23 kg/m^2 was associated with an increased risk of EAC. Statistically significant increased risk in subjects with a BMI >35. Inverse association with ≥7 units alcohol/week from wine.
Zamora-Ros et al. 2014 [135]	1992-2010<br>DK, FR, DE, GR, IT, NL, ES, SE, UK	EPIC - Cohort	339	442,143	To investigate the relationship between tea (mainly black tea) and coffee (total, caffeinated and decaffeinated) intake and EAC risk.	Validated dietary country-specific questionnaires; personal interview (GR, ES, Ragusa); short non-quantitative FFQ + 7-day dietary diary (SE); questionnaire on	No statistically significant association between the intake of tea, mainly black tea, and coffee and EAC risk.

						sociodemographic characteristics; questionnaire on physical activity and leisure time; directly measured weight and height (except in UK and FR); information on coffee and tea consumption (only in DE, NL, and UK)	
Zendehdel et al. 2008 [136]	1971-2004<br>SE	Retrospective cohort	130	336,381	To investigate the relation between smokers and users of Scandinavian moist snuff and EAC risk.	200-item questionnaire	The use of snus was not statistically significant for EAC risk. Compared to never-users of any tobacco, smokers had increased risks of EAC.

Abbreviations: **aMED**, Alternate Mediterranean Diet; **AU**, Australia; **B[a]P**, Benzo[a]pyrene; **BEAGESS**, Barrett's and Esophageal Adenocarcinoma Genetic Susceptibility Study; **BMI**, Body Mass Index; EAC, Esophageal Adenocarcinoma; **CPAI**, Cambridge Physical Activity Index; **CA**, Canada; **Ca**, calcium; **CI**, Confidence Interval; **CN**, China; **CSDs**, Carbonated Soft Drinks; **CT**, Computed Tomography; **CZ**, Czech Republic; **CONOR**: Cohort of Norway; **DE**, Germany; **DII**, Dietary Inflammation Index; **EAC**, Esophageal Adenocarcinoma; **EPIC**, European Prospective Investigation into Cancer and Nutrition; **FFQ**, Food Frequency Questionnaire; **FU**, follow-up; **GERD**, Gastroesophageal Reflux Disease; **GI**, Glycemic Index; **GL**, Glycemic Load; **GPRD**, General Practice Research Database; **GR**, Greece; **HCAs**, Heterocyclic amines; **HHHQ**, Health Habits and History Questionnaire; **HEI-2005**, Healthy Eating Index-2005; **HUNT3**: third Nord-Trøndelag Health Study; **IE**, Ireland; **LBS**, Pounds; **MetS**, Metabolic Syndrome; **Mg**, Magnesium; **NCL**, National Cancer Institute; **NL**, Netherlands, **OR**, Odds Ratio; **PL**, Poland; **RO**, Romania; **RU**, Russia; **RR**, Relative Risk; **SE**, Sweden; **SES**, Socioeconomic Status; **UK**, United Kingdom; **US**, United States; **WC**, Waist circumference; **WHR**, Waist-to-hip ratio, **y**, years.

Supplementary Table S3. Quality assessment of case-control studies, using the Newcastle-Ottawa Scale (NOS), in alphabetical order.

Author Year [Ref]	Selection				Comparability		Outcome/Exposure^			Total Score/9
	Item 1	Item 2	Item 3	Item 4	Item 5a	Item 5b	Item 6	Item 7	Item 8	
Anderson, et al. 2009 [31]	*	*	*	-	*	*	*	*	-	7
Anderson, et al. 2007 [32]	*	*	*	*	*	*	-	*	-	7
Bahmanyar, et al. 2006 [33]	*	*	*	-	*	*	*	*	*	8
Bollschweiler, et al. 2002 [34]	*	*	*	-	-	-	-	*	-	4
Chen, et al. 2002 [35]	-	-	-	-	*	*	-	*	-	3
Chen, et al. 2002 [36]	*	*	-	-	*	*	*	*	*	7
Chen, et al. 2011 [37]	*	*	*	*	*	-	-	-	-	5
Cheng, et al. 2000 [38]	*	*	*	-	*	*	-	*	*	7
Chow, et al. 1998 [39]	*	*	*	*	*	*	*	*	*	9
Corley, et al. 2008 [40]	*	-	*	*	*	*	*	*	*	8
Dai, et al. 2016 [41]	*	*	*	*	*	*	-	*	-	7
de Jonge, et al. 2006 [42]	*	*	-	*	*	*	-	*	*	7
Drahos, et al. 2016 [43]	*	*	*	*	*	*	*	*	-	8
Duan, et al. 2009 [44]	*	*	-	-	*	*	-	*	-	5
Engel, et al. 2003 [45]	*	*	*	-	*	*	-	*	*	7
Gammon, et al. 1997 [46]	*	*	*	-	*	*	-	*	*	7
Gao, et al. 1994 [47]	*	*	*	-	*	*	-	*	-	6
Garidou, et al. 1996 [48]	*	*	-	-	*	*	-	*	*	6
Hashibe, et al. 2007 [49]	*	*	-	-	*	*	-	*	*	6
Ibiebele, et al. 2012 [50]	*	*	*	-	*	*	*	*	*	8
Ibiebele, et al. 2013 [51]	*	*	*	-	*	*	*	*	*	8
Jansson, et al. 2005 [52]	*	*	*	-	*	*	*	*	-	7
Lagergren, et al. 1999 [53]	*	*	*	*	*	*	-	*	-	7
Lagergren, et al. 2000 [54]	*	*	*	-	*	*	-	*	-	6
Lagergren, et al. 2006 [55]	*	*	*	-	*	-	*	*	*	7
Lagergren, et al. 2013 [56]	*	*	*	-	*	*	-	*	-	6
Lagergren, et al. 2014 [57]	*	*	*	-	*	*	-	*	-	6
Lahmann, et al. 2014 [58]	*	*	*	-	*	*	*	-	-	6
Li, et al. 2017 [59]	*	*	*	-	*	*	*	*	-	7
Lin, et al. 2011 [60]	*	*	*	-	*	*	*	*	-	7
Lin, et al. 2014 [61]	*	*	*	-	*	*	*	*	-	7
Lin, et al. 2012 [62]	*	*	*	-	*	*	-	*	-	6
Lindblad, et al. 2005 [63]	-	*	*	*	*	*	*	*	-	7
Lu, et al. 2015 [64]	*	*	*	-	*	*	*	*	-	7
MassI, et al. 2014 [65]	*	*	-	-	*	-	*	*	-	5
Mayne, et al. 2001 [66]	*	*	*	-	*	*	*	*	*	8
Mayne, et al. 2006 [67]	*	*	*	-	*	*	-	*	-	6
Mulholland, et al. 2011 [68]	*	*	*	*	*	*	-	*	*	8
Murphy, et al. 2010 [69]	*	*	*	*	*	*	-	*	-	7
Navarro Silvera, et al. 2014 [72]	*	*	*	-	*	*	*	*	-	7

Navarro Silvera, et al. 2008 [70]	*	*	*	-	*	*	*	*	-	7
Navarro Silvera, et al. 2011 [71]	*	*	*	-	*	*	*	*	-	7
O'Doherty, et al. 2010 [73]	*	*	*	*	*	*	-	*	-	7
O'Doherty, et al. 2011 [74]	*	*	*	*	*	*	-	*	-	7
Olsen, et al. 2011 [75]	*	*	*	-	*	*	-	*	-	6
Pandeya, et al. 2008 [76]	*	*	*	-	*	*	-	*	-	6
Pandeya, et al. 2009 [77]	*	*	*	-	*	*	-	*	-	6
Pandeya, et al. 2010 [78]	*	*	*	-	*	*	-	*	-	6
Petrick, et al. 2015 [79]	*	*	*	-	*	*	*	*	*	8
Pohl, et al. 2013 [80]	*	*	-	*	*	*	-	*	-	6
Ryan, et al. 2006 [81]	*	*	*	-	*	*	-	*	-	6
Sharp, et al. 2013 [82]	*	-	*	*	*	*	*	*	-	7
Terry, et al. 2000 [83]	*	*	*	-	*	*	-	*	*	7
Terry, et al. 2001 [84]	*	*	*	-	*	*	-	*	-	6
Terry, et al. 2003 [85]	*	*	*	-	*	*	-	*	-	6
Thrift, et al. 2014 [86]	*	-	*	*	*	*	*	*	-	7
Tzonou, et al. 1996 [87]	*	*	-	-	*	*	*	*	*	7
Veugelers, et al. 2006 [88]	*	*	-	-	*	*	-	*	-	5
Vigen, et al. 2006 [89]	*	*	*	-	*	*	*	*	-	7
Ward, et al. 1997 [90]	*	*	*	-	*	*	-	*	-	6
Ward, et al. 2012 [92]	*	*	*	-	*	*	-	*	-	6
Ward, et al. 2008 [91]	*	*	*	-	*	*	-	*	*	7
Whiteman, et al. 2008 [93]	*	*	*	-	*	*	-	*	-	6
Wolfgarten, et al. 2001 [94]	*	*	*	-	-	-	*	*	-	4
Wu, et al. 2007 [96]	*	*	*	*	*	*	-	*	-	7
Wu, et al. 2001 [95]	*	*	*	*	*	*	-	*	-	7

^based on study design. For case-control the NOS assessed the exposure, for cross-sectional and cohort studies the outcome.

Supplementary Table S4. Quality assessment of cohort studies, using the Newcastle-Ottawa Scale (NOS), , in alphabetical order.

Author Year [Ref]	Selection				Comparability		Outcome/Exposure^			Total
	Item 1	Item 2	Item 3	Item 4	Item 5a	Item 5b	Item 6	Item 7	Item 8	Score/9
Abnet, et al. 008 [97]	*	*	*	*	*	*	*	-	*	8
Allen, et al. 2009 [98]	*	*	-	*	*	*	*	-	*	7
Carman, et al. 2009 [99]	*	*	*	*	*	*	*	-	*	8
Cook, et al. 2013 [100]	*	*	-	*	*	*	*	*	-	7
Cross, et al. 2011 [101]	*	*	*	*	*	*	*	*	-	8
Dawsey, et al. 2014 [102]	*	*	*	*	*	*	*	*	-	8
De Jonge, et al. 2007 [103]	*	-	*	-	*	*	**	*	n.a.	7
Engeland, et al. 2004 [104]	*	*	*	*	*	*	*	*	*	9
Freedman, et al. 2007 [105]	*	*	-	*	*	*	*	-	*	7
Freedman, et al. 2007 [106]	*	*	*	-	*	*	*	-	*	7
Gatenby, et al. 2008 [107]	*	*	*	*	*	-	*	*	-	7
Gonzalez, et al. 2006 [108]	*	*	*	-	*	*	*	-	*	7
Gonzalez, et al. 2006 [109]	*	*	*	-	*	*	*	-	*	7
Hardikar, et al. 2013 [110]	*	*	*	*	*	*	*	*	*	9
Huerta, et al. 2010 [111]	*	*	*	-	*	*	*	*	*	8
Jakszyn, et al. 2013 [112]	*	*	*	*	*	*	*	*	*	9
Ji, et al. 2016 [113]	*	-	*	-	*	*	*	*	*	7
Keszei, et al. 2012 [114]	*	*	*	-	*	*	*	*	*	8
Keszei, et al. 2013 [115]	*	*	*	*	*	*	*	*	*	9
Levi, et al. 2013 [116]	*	*	-	-	*	*	*	*	*	7
Li, et al. 2013 [117]	*	*	-	*	*	*	*	*	-	7
Lin, et al. 2015 [118]	*	*	*	*	*	*	*	*	-	8
Merry, et al. 2007 [119]	*	*	*	*	*	*	*	*	*	9
O'Doherty, et al. 2012 [120]	*	*	*	*	*	*	*	*	-	8
O'Doherty, et al. 2012 [121]	*	*	-	-	*	*	*	*	*	7
Petrick, et al. 2017 [122]	*	*	-	*	*	*	*	*	*	8
Reeves, et al. 2007 [123]	*	*	-	*	*	*	*	-	-	6
Ren, et al. 2010 [124]	*	*	*	*	*	*	*	-	*	8
Samanic, et al. 2006 [125]	*	*	*	-	*	*	*	*	*	8
Sanikini, et al. 2020 [126]	*	*	*	*	*	*	*	*	*	9
Steevens, et al. 2010 [127]	*	*	*	*	*	*	*	*	*	9
Steevens, et al. 2010 [128]	*	*	*	*	*	*	*	*	*	9
Steevens, et al. 2011 [129]	*	*	*	*	*	*	*	*	*	9
Steffen, et al. 2009 [130]	*	*	*	*	*	*	*	-	*	8
Steffen, et al. 2015 [131]	*	*	*	*	*	*	*	*	*	9
Vermeulen, et al. 2013 [132]	*	*	*	*	*	*	*	*	*	9
Xiao, et al. 2014 [134]	*	*	-	*	*	*	*	*	*	8

Yates, et al. 2014 [133]	*	*	*	*	*	-	*	*	*	8
Zamora-Ros, et al. 2014 [135]	*	*	*	*	*	*	*	*	*	9
Zendehdel, et al. 2008 [136]	*	*	*	-	*	*	*	*	*	8

^based on study design. For case-control the NOS assessed the exposure, for cross-sectional and cohort studies the outcome.
